# Pre‐clinical evidences for the efficacy of tryptanthrin as a potent suppressor of skin cancer

**DOI:** 10.1111/cpr.12710

**Published:** 2019-10-30

**Authors:** Mohan Shankar G., Vijai V. Alex, Amrutha Nisthul A., Smitha V. Bava, Sankar Sundaram, Archana P. Retnakumari, Sadasivan Chittalakkottu, Ruby John Anto

**Affiliations:** ^1^ Division of Cancer Research Rajiv Gandhi Centre for Biotechnology Thiruvananthapuram Kerala India; ^2^ Research Scholar Manipal Academy of Higher Education Manipal Karnataka India; ^3^ Department of Biotechnology and Microbiology Kannur University Kannur Kerala India; ^4^ Department of Biotechnology University of Calicut Calicut Kerala India; ^5^ Department of Pathology Government Medical College Kottayam Kerala India

**Keywords:** multistage carcinogenesis model, pre‐clinical study, skin cancer, Tryptanthrin

## Abstract

**Objective:**

Clinical trials have demonstrated the efficacy of indigo naturalis, a traditional Chinese medicine ingredient, against psoriasis, a skin disease characterized by keratinocyte hyperproliferation and inflammation. The present study investigates the efficacy of tryptanthrin, a bioactive compound in indigo naturalis, against non‐melanoma skin cancer (NMSC) and the signalling events involved.

**Methods:**

Efficacy of tryptanthrin against NMSC was assessed using DMBA/PMA‐induced skin carcinogenesis model in *Swiss albino* mice. Immunostaining for PCNA and ki‐67 was used to mark proliferating cells in tissues. Haematoxylin and eosin staining and toluidine staining were employed to assess inflammation, and TUNEL assay was used to detect apoptosis in tissues. The signalling events were evaluated using Western blot, imunohistochemistry and immunofluorescence staining. MTT assay and clonogenic assay were performed to assess the viability and proliferation of cancer cells, in vitro.

**Results:**

In mice, topical application of tryptanthrin suppressed skin carcinogenesis. It attenuated inflammation, impeded the proliferation of hair follicle (HF) cells and suppressed the activation of β‐catenin, a major driver of HF cell proliferation. Additionally tryptanthrin suppressed the activation of ERK1/2 and p38, both of which promote β‐catenin activation and lowered the expression of c‐Myc and cyclin‐D1. Tryptanthrin suppressed the proliferation of the human NMSC cell line, A431 and abrogated EGF‐induced activation of β‐catenin and subsequent cytoskeletal rearrangement.

**Conclusion:**

The study demonstrates with molecular evidence that tryptanthrin is an effective suppressor of NMSC.

## 1 INTRODUCTION

Non‐melanoma skin cancer (NMSC) is the most frequently diagnosed cancer globally.^1^ According to the World Health Organization, one in every three cancers diagnosed is a skin cancer, with basal cell carcinomas (BCCs) and squamous cell carcinomas (SCCs) being the most common subtypes.^2^ Currently used therapeutic agents against NMSC, namely, 5‐fluorouracil and imiquimod, inflict deleterious side effects to normal cells.^3^, ^4^ At this juncture, it is imperative to identify novel non‐toxic compounds that suppress skin tumourigenesis.

Tumourigenesis is a multi‐factorial process, altering multiple signalling pathways, which confer the mutated cells with increased potential to proliferate, evade apoptosis and expand.^5^ Therefore, using compounds that can hinder multiple pro‐tumourigenic factors is an attractive strategy, as it exerts a multi‐faceted approach that effectively curbs tumourigenesis. The possibility of using natural products as anti‐cancer agents is being explored for devising new treatment modalities. Many of these compounds are currently being used as mainstream therapeutic agents against cancers of various origins.

Indigo naturalis is a herbal preparation used in Chinese traditional medicines. Clinical trials have revealed its effectiveness in curing psoriasis, an inflammatory disease of the skin showing hyperproliferation of keratinocytes.^6^, ^7^ One of the major bioactive components of indigo naturalis is tryptanthrin, an indoloquinazoline alkaloid reported to have anti‐cancer and anti‐inflammatory activity.^8^, ^9^, ^10^ Moreover, we have identified tryptanthrin as one of the bioactive compounds present in DW‐F5, an anti‐cancer fraction from *Wrightia tinctoria* effective against melanoma.^11^ However, there are no detailed reports on the effect of tryptanthrin against any type of skin cancer. Experimental evidences suggest a link between chronic inflammation and skin cancer.^12^, ^13^ Considering the aforementioned leads, we hypothesized that tryptanthrin could be an effective molecule against NMSC. The present study aims to assess the efficacy of tryptanthrin against NMSC and elucidate the molecular mechanism involved.

## 2 MATERIALS AND METHODS

### 2.1 Reagents and antibodies

DMBA, PMA, antibodies against β‐actin, vinculin and horseradish peroxidase (HRP)‐conjugated secondary antibodies were obtained from Sigma‐Aldrich. Tryptanthrin was purchased from Accela ChemBio. DAPI (4′,6‐Diamidino‐2‐phenylindole dihydrochloride), antibodies against p‐JNK, JNK1, p38, STAT3, ERK1/2, β‐catenin and cyclin‐D1 were purchased from Santacruz Biotechnology. Antibodies against p‐Akt, p‐p42/44, p‐p38, PCNA, p‐STAT3, p‐c‐JUN, Lamin B1 and c‐Myc were purchased from Cell Signaling Technologies. Immobilon Western Chemiluminescent HRP Substrate was purchased from Millipore. Antibody against Ki‐67 was purchased from Thermo Fisher Scientific. All other reagents were procured from Sigma‐Aldrich, unless otherwise mentioned.

### 2.2 Preparation and characterization of liposomal formulation of tryptanthrin

For preparing liposomal tryptanthrin, 5 mg of tryptanthrin was dissolved in chloroform and methanol at 3:1 v/v along with 45 mg of phosphatidylcholine and 5.8 mg of cholesterol and the mixture was evaporated in a rotary vacuum evaporator (Buchi) followed by suspending the mixture in 3 mL of PBS (pH 7.4). The suspension was sonicated using an ultra‐sonicator prior to application. The percentage encapsulation of tryptanthrin was obtained by dispersing liposomal tryptanthrin in DMSO after removing the unbound tryptanthrin by two washes in PBS (pH 7.4). Later, percentage of tryptanthrin loaded in liposome was calculated from a standard graph plotted by serially diluting 1 mg/mL of tryptanthrin and recording the absorbance at 390 nm.

### 2.3 In vivo studies

The chemoprevention studies using liposomal tryptanthrin formulation were carried out in *Swiss albino* mice, according to the prescribed guidelines, which were approved by the Institutional Animal Ethical Committee, RGCB.

### 2.4 Carcinogenesis experiments

For all carcinogenesis experiments, 100 µg of DMBA was applied topically on female *S albino* mice (7‐8 weeks old), once a week for 2 weeks, followed by application of PMA twice weekly for 16 weeks (D + P). For assessing the potential of tryptanthrin in suppressing the promotion stage of carcinogenesis, liposomal tryptanthrin, at doses of 0.5 mg or 1 mg, was applied topically on animals, 1h before each application of PMA (D + P + T [0.5 mg] or D + P + T [1 mg]). To confirm the pharmacological safety of tryptanthrin, one group of animals were topically treated only with 1mg of liposomal tryptanthrin (IAEC/191). For assessing the extend of proliferation of cells and the status of proliferative signals, DMBA application was done once a week for 2 weeks, followed by five applications of 5 μg of PMA, within a period of 2 weeks. Tryptanthrin (0.5 mg or 1 mg) was applied before each application of PMA. The last dose of PMA was applied 24 hours after the fourth application, and animals were sacrificed 6 hours after final application. One group was treated with 1 mg of tryptanthrin to assess its effect on normal skin (IAEC/550).

### 2.5 Induction of hyperplasia

Hyperplasia was induced according to the protocol reported.^14^ Approximately, 5 μg of PMA was applied on the dorsal region of skin twice weekly (on alternate days), for 1 week. Animals were sacrificed 6 hours after the last dose of PMA. In the treatment group, tryptanthrin (0.5 mg and 1 mg) was applied 1 hour prior to each application of PMA. Another group of animals, which received 1 mg of tryptanthrin alone were also included in the study (IAEC/551).

### 2.6 Protein isolation and immunoblotting

Protein isolation from tissue/cells and immunoblotting were performed using protocols as mentioned elsewhere.^15^ Skin/tumour was homogenized in ice‐cold lysis buffer containing 1 mol/L Tris (pH7.4), NaCl (5 mol/L), EDTA (0.5 mol/L), DTT (0.1 mol/L), TritonX100, protease inhibitors and sodium orthovanadate (4 mmol/L). The homogenate was kept in ice, subjected to intermittent vortexing for 30 minutes at 5 minutes interval, followed by centrifugation at 17982 *g* for 10 minutes. The supernatant was mixed with 5X sample buffer, boiled at 95°C for 5 minutes and stored at −70°C until use.

### 2.7 Isolation of nuclear protein

Nuclear proteins were isolated by homogenizing tissues in buffer A (500 μL of buffer for ~100 mg tissue) containing HEPES (10 mmol/L), KCl.

(1.5 mmol/L), MgCl_2_ (10 mmol/L), DTT (0.5 mmol/L), IGEPAL (0.1%), PMSF (0.5 mmol/L) and centrifuged at 20854 *g* at 4°C for 10 minutes. The supernatant was stored as the cytoplasmic fraction. The pellet was again washed with buffer A, to make sure that all the cytoplasmic contents are separated from the nuclear fraction. To the pellet, 100 μL of ice‐cold buffer B containing HEPES (20 mmol/L), glycerol (25%), NaCl (420 mmol/L), MgCl_2_ (1.5 mmol/L), DTT (0.5 mmol/L), EDTA (0.2mmol/L), PMSF (0.5 mmol/L) and leupeptin (2 μg/mL) was added, mixed and incubated for 2 hours with intermittent vortexing at every 10 minutes. The mixture was centrifuged at 17982 *g* for 30 minutes at 4°C after which, the supernatant (nuclear fraction) was stored at −70°C until use.

### 2.8 Histopathology

Histopathology of skin tissues was performed using haematoxylin and eosin staining (H&E) as mentioned elsewhere.^15^

### 2.9 Immunohistochemistry

Immunohistochemical analysis of formalin‐fixed, paraffin‐embedded/cryosectioned skin/tumour tissues, were performed using Mouse on Mouse Elite Peroxidase Kit (Vector Labs). Alternatively, tissues were subjected to direct/indirect immunofluorescence studies. Bright field and fluorescent images were captured using Leica DMi8 microscope and Nikon eclipse confocal microscope, respectively.

### 2.10 Toluidine blue staining

Paraffin‐embedded sections of mouse skin were subjected to de‐paraffinization and hydration followed by staining with a freshly prepared working solution of toluidine blue for 3 minutes. The sections were washed 3 times in distilled water and subjected to dehydration using 70% and 95% isopropanol, cleared in Xylene and mounted using DPX mountant.

### 2.11 Cell lines

A431 cells were procured from NCCS, Pune. Foreskin fibroblasts were a gift from Dr Bharat B Agarwal. Cell lines were maintained in DMEM supplemented with FBS (10%) and incubated in 5% CO_2_ at 37°C.

### 2.12 MTT assay

Two thousand cells were seeded in a 96‐well plate and incubated overnight followed by incubation with different concentrations of the compounds for 72 hours before assessing the viability using 20% MTT solution in DMEM.

### 2.13 Clonogenic assay

For assessing the colony‐forming potential of A431 cells, 500 cells were seeded/well in a 12‐well plate, incubated overnight and were exposed to different concentrations of the compounds for 72 hours. The cells were then allowed to proliferate in fresh media for 72 hours prior to fixation and staining with crystal violet.

### 2.14 Immunofluorescence

Cells were seeded in coverslips in a 24‐well plate, incubated overnight and were incubated with tryptanthrin and EGF for 24 hours. Cells were then fixed using methanol‐EDTA solution, blocked and incubated overnight with primary antibodies followed by incubation with fluorescent‐tagged secondary antibodies. Then, the cells were stained with DAPI and mounted on slides.

### 2.15 Molecular docking studies

The binding affinity of tryptanthrin towards human ERK2 was analysed using molecular docking studies. All in silico analyses were conducted using the program, Schrodinger suite (Schrödinger, LLC, 2018). The crystal structure of ERK2 in complex with the selective ERK inhibitor FR180204 (PDB ID: http://www.rcsb.org/pdb/search/structidSearch.do?structureId=1TVO) was downloaded from Protein Data Bank (PDB).The molecular coordinates of tryptanthrin were extracted from PubChem (CID: 73549). Structure correction of ERK2 was performed prior to docking run using the module, “Protein preparation wizard”. Water molecules with less than three H‐bonds to non‐waters were removed, missing hydrogen atoms were added, H‐bond network was optimized and minimization of the structure was performed up to 0.3 Å using OPLS‐3e force field. Ligand preparation was performed using the module, LigPrep. Different tautomeric and ionization states corresponding to pH 7 ± 2 were generated and the conformers were docked onto the 20 Å × 20 Å × 20 Å receptor grid in standard precision (SP) method using OPLS‐3e force field. The binding free energies of protein‐ligand complexes were predicted using Prime MMGBSA.

### 2.16 Statistical methods

For the carcinogenesis experiments, the statistical significance of the observations was assessed by Student's two‐tailed, unpaired *t* test. *P*‐value <.05 was considered significant. Statistical analysis was done using GraphPad Prism.

## 3 RESULTS

### 3.1 Tryptanthrin suppresses DMBA/PMA‐induced skin carcinogenesis

**Figure 1:**
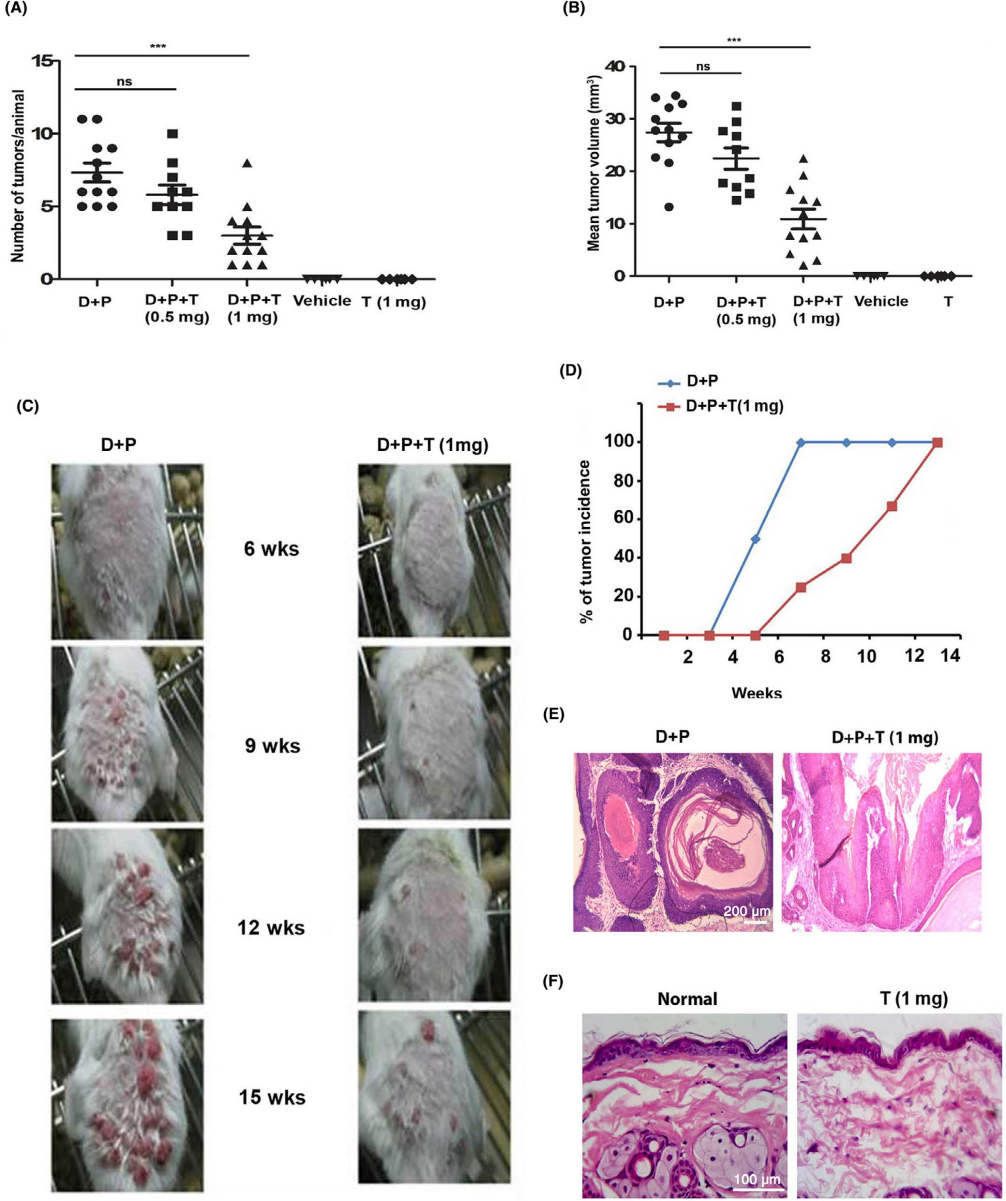
Tryptanthrin suppresses DMBA/PMA‐induced skin carcinogenesis**.** Animals were topically applied with DMBA followed by PMA, twice weekly for 16 wk. Animals in the treatment group (D + P + T) received 0.5 mg or 1 mg of liposomal tryptanthrin 1 h before PMA application while the untreated group (D + P) received blank liposome. Animals in the vehicle group were applied topically with acetone only. Animals in the fifth group received tryptanthrin at a dose of 1 mg throughout the experiment. A, Scatter plot showing the number of tumours in each animal in D + P (n = 12), D + P + T‐0.5 mg (n = 10), D + P+T‐1 mg (n = 12), vehicle (n = 6) and liposomal tryptanthrin. B, Scatter plot showing mean tumour volume on each animal in the respective groups. C, Photograph showing tumour growth in untreated and tryptanthrin‐treated groups. D, Tumour incidence at each week in animals from the untreated and treated groups. E, H&E staining of representative tumours from the respective groups. Scale bar, 200 μm. F, H&E staining of the normal skin and the skin treated with 1 mg of tryptanthrin for 16 wk. Scale bar, 100 μm. Statistical analysis of tumour number and size between the groups were done by Student's two‐tailed, unpaired *t* test. *** indicates *P* < .0001 and ns indicates no significance

To study whether tryptanthrin can suppress skin cancer, multistage carcinogenesis model using *Swiss albino* mice, in which tumours were induced by topical application of DMBA followed by PMA, was used. Two different concentrations of liposomal tryptanthrin (0.5 mg and 1 mg), which exhibited more than 90% encapsulation efficiency (Figure S1) were used. These doses were chosen based on previous reports evaluating the efficacy of other phytochemicals in suppressing skin carcinogenesis.^14^ We observed 60.1% reduction in tumour size (27.41 ± 1.77 mm^3^ in untreated vs 10.93 ± 1.89 mm^3^ in tryptanthrin treated) and 59% reduction in tumour multiplicity (7.3 ± 0.64 in untreated vs 3.00 ± 0.59 in tryptanthrin treated) in the animals treated with 1 mg of liposomal tryptanthrin (D + P + T) (Figure 1A‐C). Moreover, while 100% of the animals in the untreated group developed tumours within 7 weeks of PMA application, it took 13 weeks for the animals in the tryptanthrin‐treated group to reach 100% tumour incidence. It was also interesting to note that, at the 7th week there was only 20% tumour incidence in the tryptanthrin‐treated group (Figure 1D). The development of micro‐invasive carcinoma was histopathologically confirmed in the tumours of animals in D + P group (Figure 1E). Tumours from tryptanthrin‐treated (1 mg) animals were graded as papilloma with mild dysplasia. The application of 1 mg liposomal tryptanthrin did not cause any pathological changes to normal skin, confirming the biological safety of the compound (Figure 1F). Hence, this experiment clearly demonstrates that tryptanthrin is an excellent suppressor of skin carcinogenesis.

### 3.2 Tryptanthrin suppresses the proliferation of hair follicle cells and inflammation induced by DMBA/PMA

**Figure 2:**
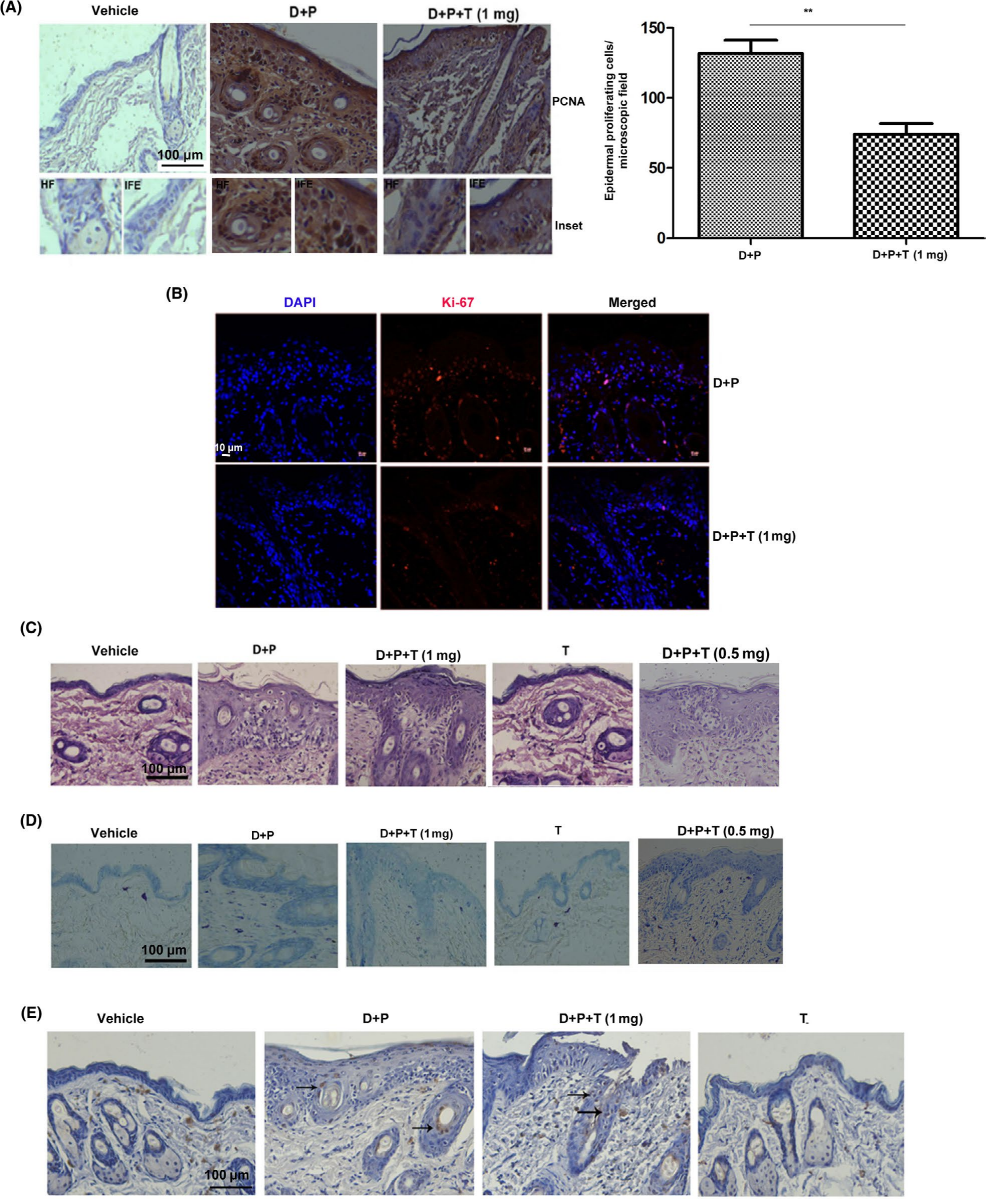
Tryptanthrin suppresses the proliferation of hair follicle cells and inflammation induced by DMBA/PMA. Animals were treated with DMBA (100 μg in 100 μl acetone) followed by five applications of PMA (5 μg in 100 μl acetone) that spanned over 2 wk. Liposomal tryptanthrin or blank liposome was applied 1 h prior to each application of PMA. Animals were sacrificed 6 h after the final application of PMA and skin was excised out for further studies. A, PCNA staining on tissue sections from respective groups. Inset shows PCNA staining in hair follicle and inter‐follicular epidermis. Graph shows proliferating cells/microscopic field. Scale bar, 100 μm. * indicates *P* < .05. n = 4. B, Ki‐67 staining on tissue sections from different groups. Scale bar, 10μm. C, H&E staining on tissue sections showing infiltration of inflammatory cells into the epidermis. Scale bar, 100 μm. D, Toluidine staining to assess mast cell infiltration on skin sections from different groups. Scale bar, 100 μm. *** indicates *P* < .001. n = 4 E, TUNEL assay on the skin from the respective groups. Arrows show apoptotic cells. Scale bar, 100 μm

Proliferation and expansion of hair follicle (HF) cells are considered as one of the principal drivers of skin carcinogenesis.^16^ Chronic application of PMA impels proliferation and expansion of initiated/mutated HF stem cell into the basal and supra‐basal layers of the epidermis leading to the development of papilloma.^17^ We conducted a short‐term experiment to assess the potential of tryptanthrin to quell DMBA/PMA‐induced proliferation and expansion of hair follicle cells. Mice were treated with DMBA followed by five applications of PMA (5 μg) over a period of two weeks. In the treatment group, tryptanthrin (0.5 mg or 1 mg) was applied 1 hour before each application of PMA. The skin was dissected from respective groups 6 hours post‐final application of PMA. Analysis of the expression status of proliferation markers, PCNA and ki‐67 in skin of various experimental groups by immunohistochemistry revealed that DMBA/PMA application drastically increases the expression of these markers in the cells of the hair‐follicle, basal and supra‐basal regions. Interestingly, application of tryptanthrin prior to DMBA/PMA lowered the expression of these markers, with the hair follicle cells showing much less staining compared with inter‐follicular epidermal (IFE) cells (Figure 2A,B). Haematoxylin and eosin (H&E) staining of skin from different experimental groups revealed that tryptanthrin is an excellent inhibitor of inflammation as indicated by low infiltration of inflammatory cells (neutrophils and lymphocytes) into the epidermis in tryptanthrin‐treated skin (D + P + T) compared with the untreated (D + P; Figure 2C). The anti‐inflammatory role of tryptanthrin was further confirmed by toluidine staining, which demonstrated the potential of tryptanthrin in suppressing DMBA/PMA‐induced mast cell infiltration (Figure 2D, Figure S2A). However, we did not observe a significant change in the level of apoptosis either in the skin or tumour of mice treated with tryptanthrin, as assessed by TUNEL assay (Figure 2E, Figure S2B, S2C). Hence, these results clearly indicate that tryptanthrin hampers the promotion stage of skin carcinogenesis by restricting the expansion of mutated hair follicle cells and by suppressing inflammation, but not by inducing apoptosis.

### 3.3 Tryptanthrin hinders DMBA/PMA‐induced β‐catenin activation

**Figure 3:**
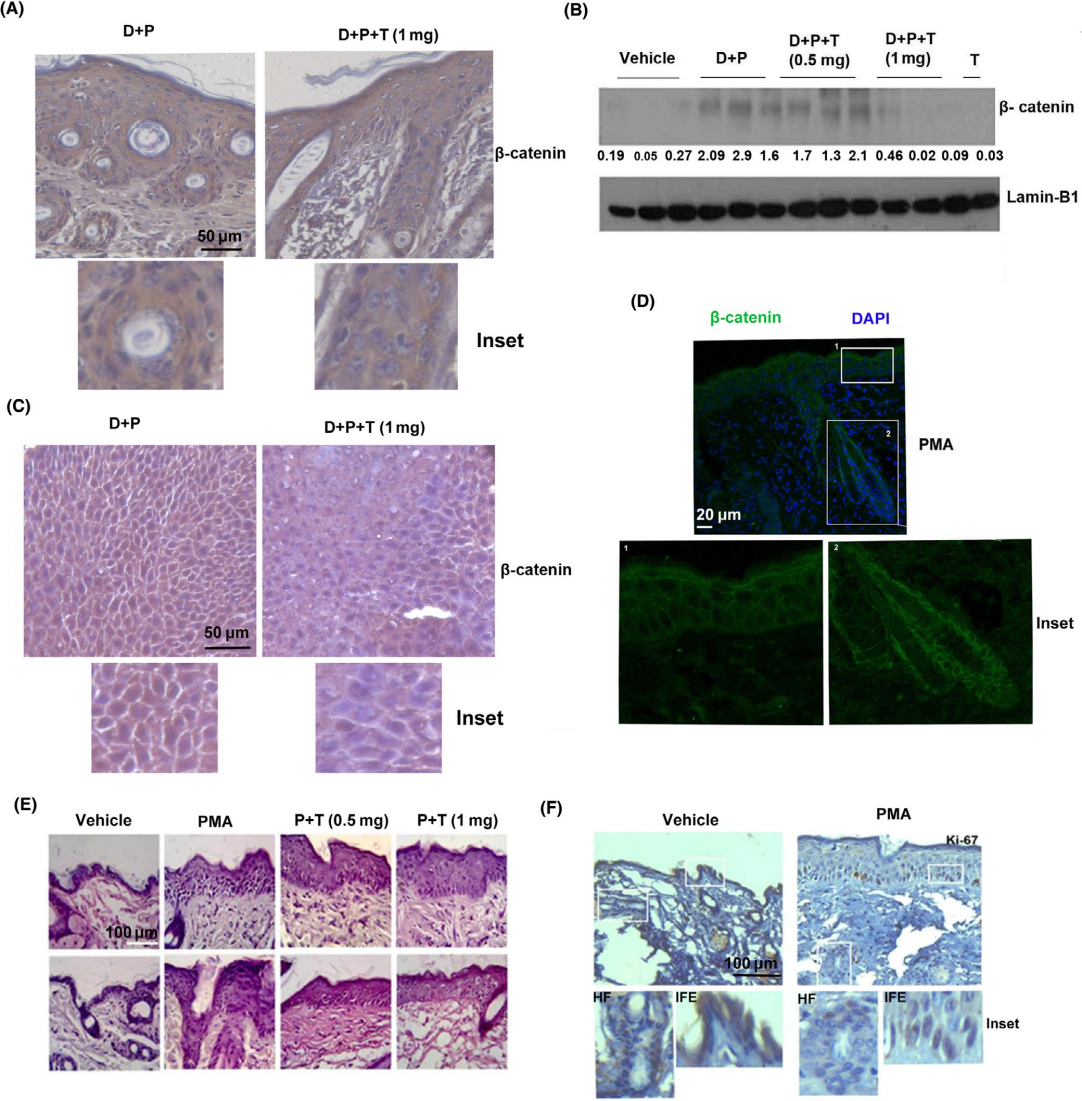
Tryptanthrin hinders DMBA/PMA‐induced β‐catenin activation in mouse skin. A, Immunohistochemical analysis of β‐catenin in the skin of untreated (D + P) and tryptanthrin‐treated animals (D + P + T). Inset shows staining for β‐catenin in hair follicle cells. Scale bar, 50 μm. B, Immunoblot of nuclear protein isolated from animals from the respective groups showing nuclear translocation of β‐catenin. C, Immunohistochemical staining of β‐catenin in tumours from the respective groups. Scale bar, 50 μm. D, Immunofluorescent staining of β‐catenin in the skin of animals treated with PMA alone. Scale bar, 20 μm. Inset shows staining for β‐catenin in hair follicle cells and inter‐follicular epidermal cells. E, Images show H&E‐stained sections of skin from the treated (P + T) and untreated (PMA) groups. Scale bar, 100 μm. F, Immunostaining of ki‐67 in PMA‐treated skin

Activation of β‐catenin is essential for the expansion of hair‐follicle stem cells during skin carcinogenesis.^18^ As our results establish the potential of tryptanthrin in suppressing DMBA/PMA‐induced expansion of hair follicle cells, we compared the activation status of β‐catenin in mouse skin from various experimental groups of the short‐term experiment. Immunohistochemical analysis displayed an elevated nuclear localization of β‐catenin in the hair follicle cells of the untreated group, compared to those treated with tryptanthrin (Figure 3A). Furthermore, to assess whether β‐catenin is activated in all the proliferating hair follicle cells, we conducted double immunofluorescence study, which demonstrated that most of the proliferating cells in the D + P group exhibits β‐catenin activation. However, in the group treated with tryptanthrin, β‐catenin was not active in hair follicle cells and the expression of PCNA was relatively low (Figure S3A). Immunoblot analysis of nuclear protein isolated from the respective experimental groups confirms the potential of tryptanthrin to inhibit the nuclear translocation of β‐catenin (Figure 3B, Figure S3B). Additionally, skin tumours harvested from the animals treated with tryptanthrin in the multistage skin carcinogenesis model (long‐term) had significantly less nuclear β‐catenin compared with those harvested from untreated animals (Figure 3C, Figure S3C). To test the efficacy of the compound to modulate β‐catenin‐independent proliferation, we assessed the potency of the compound in suppressing epidermal hyperproliferation, which is a β‐catenin‐independent process.^19^ Epidermal hyperplasia was induced in the animals by the topical application of two doses of PMA on alternate days and the skin was excised out 6 hours post‐application of the second dose. In the treatment group, tryptanthrin at doses of 0.5 mg or 1 mg was applied 1 hour prior to each application of PMA. Application of two doses of PMA on uninitiated skin (normal skin) did not induce activation of β‐catenin in the inter‐follicular epidermal or hair follicle cells (Figure 3D) though it caused epidermal hyperplasia. However, application of tryptanthrin prior to PMA (P + T) did not suppress PMA‐induced epidermal hyperplasia (Figure 3E). To confirm that the HF cells did not contribute to the observed hyperplasia, immunostaining of ki‐67 was executed. We observed that the proliferation marker, ki‐67 did not show significant staining in HF cells whereas the cells in the inter‐follicular epidermal region showed appreciable staining (Figure 3F). These results illustrate the efficacy of tryptanthrin in abrogating β‐catenin‐dependent proliferation of skin cancer cells.

### 3.4 Tryptanthrin is a potent inhibitor of cell survival signals induced by DMBA/PMA

**Figure 4:**
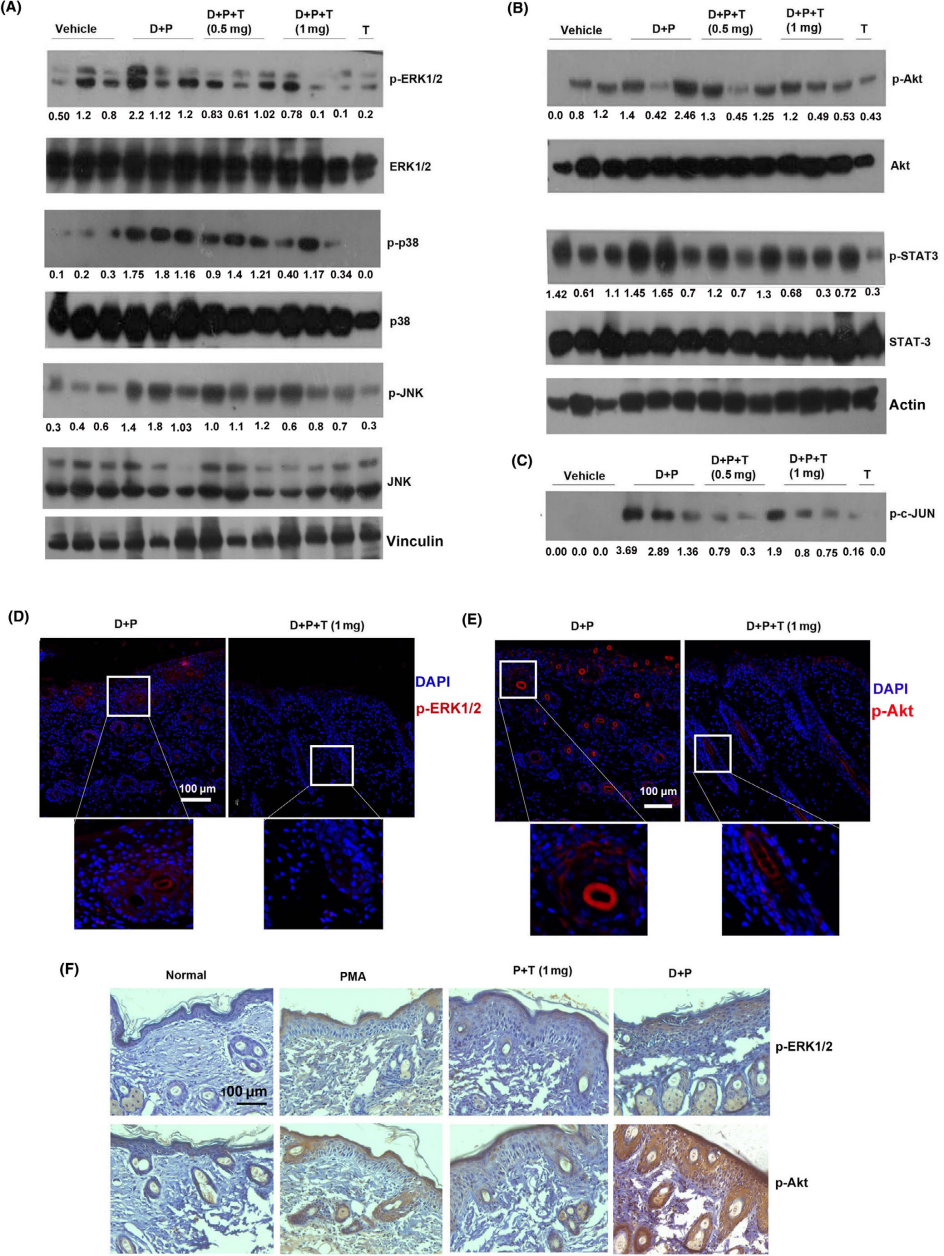
Tryptanthrin is a potent inhibitor of cell survival signals induced by DMBA/PMA. A, Immunoblot analysis showing extent of phosphorylation of MAP kinases in skin isolated from animals of various groups from the short‐term experiment. B, Immunoblots showing the phosphorylation and relative expression of Akt, STAT3. C, Immunoblot analysis of c‐Junin nuclear fraction of skin from the respective groups. D, Immunofluorescent staining of p‐ERK1/2 tryptanthrin‐treated (D + P + T) and untreated (D + P) groups. Scale bar, 100 μm. Inset shows staining for p‐ERK1/2 in hair follicle cells. E, Immunofluorescent staining of p‐Akt tryptanthrin‐treated (D + P+T) and untreated (D + P) groups. Scale bar, 100 μm. Inset shows staining of p‐Akt in hair follicle cells. F, Immunohistochemical analysis of p‐ERK1/2 and p‐Akt in skin from the respective groups. Scale bar, 100 μm

To further investigate the molecular events regulating the chemopreventive efficacy of tryptanthrin, the activation status of key signalling pathways that contributes to PMA‐induced promotion of skin carcinogenesis were analysed. Activation of MAPK and Akt are mainstay events in skin carcinogenesis induced by DMBA/PMA.^20^, ^21^ Furthermore, MAPKs‐ERK1/2 and p38 are known to operate upstream to β‐catenin in skin cancer.^22^, ^23^ Immunoblot analysis of total protein isolated from the dorsal skin of animals revealed that tryptanthrin could accomplish remarkable attenuation of DMBA/PMA‐induced activation of MAP kinases, especially ERK1/2 (Figure 4A, Figure S4A). However, tryptanthrin could achieve only mild attenuation of Akt phosphorylation (Figure 4B, Figure S4A). Furthermore, DMBA/PMA‐induced activation of STAT3, another important event involved in the promotion of skin carcinogenesis,^24^ was abrogated by tryptanthrin (Figure 4B). To assess the phosphorylation status of c‐Jun, a component of AP‐1 transcriptional complex, which is reported to get phosphorylated by ERK1/2 at ser 63 and 73,^25^, ^26^ we reprobed the membrane used for detecting β‐catenin, with phospho‐c‐Jun antibody. Our result demonstrates a drastic decrease in the phosphorylation status of c‐Jun in skin isolated from tryptanthrin‐treated mice compared with the untreated ones (Figure 4C, Figure S4A). Immunohistochemical analysis of p‐ERK1/2 and p‐Akt revealed that, as in the case of β‐catenin, these are predominantly located in and near to the hair follicles of skin applied with DMBA/PMA. Interestingly, tryptanthrin could successfully abrogate the activation of both these proteins in the hair follicles (Figure 4D,E). Hence, the above results clearly demonstrate that tryptanthrin‐mediated abrogation of MAPK and Akt pathways in the HF cells halts their expansion. Further analysis by immunohistochemistry revealed that compared with normal skin, the hyperplastic (treated with PMA alone) skin exhibits more staining for both p‐Akt and p‐ERK. As observed in the short‐term experiment, tryptanthrin suppresses PMA‐induced phosphorylation of Akt and ERK in hyperplasia too but the activation of ERK is less in hyperplastic skin compared with that in DMBA/PMA‐treated skin (Figure 4F, Figure S4B). This could be the reason for the inefficacy of tryptanthrin in preventing epidermal hyperplasia. Taken together, these results clearly indicate the pivotal role of β‐catenin and its associated signalling pathways in regulating the anti‐cancer potential of tryptanthrin.

### 3.5 Tryptanthrin suppress DMBA/PMA‐induced expression of Cyclin‐D1 and c‐Myc

**Figure 5:**
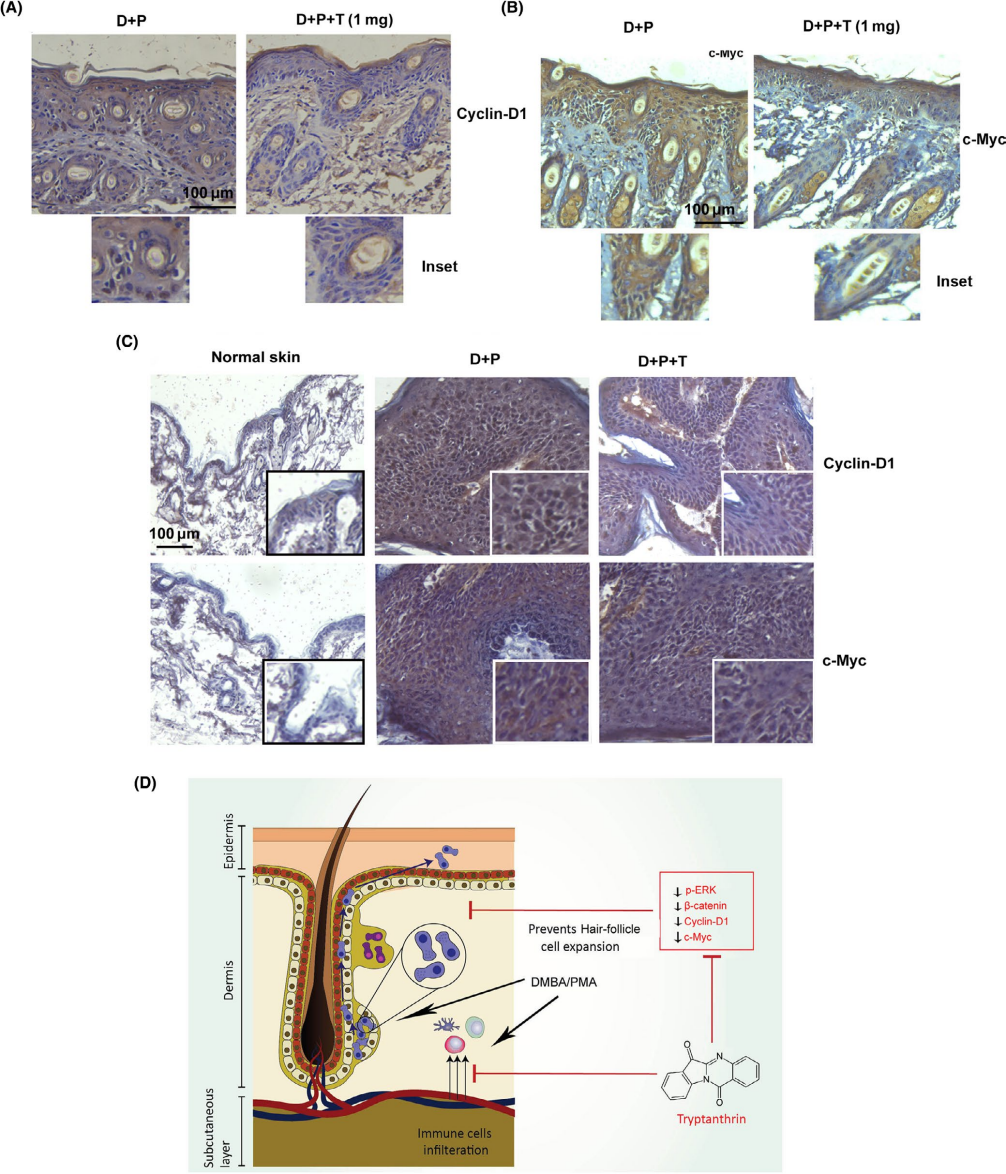
Tryptanthrin suppress DMBA/PMA‐induced expression of Cyclin‐D1 and c‐Myc (A) Immunostaining of cyclin‐D1 in skin of animals from the untreated (D + P) and treated groups (D + P + T). Scale bar, 100 μm. Inset shows staining of cyclin‐D1 in hair follicle cells. B, Immunostaining for c‐Myc in skin from the untreated (D + P) and treated group (D + P+T). Inset shows staining of c‐Myc in hair follicle cells. Scale bar, 100 μm (C) Immunohistochemical staining of c‐myc and cyclin‐D1 in normal skin and tumours from untreated and tryptanthrin‐treated animals. Scale bar, 100 μm. D, Graphical representation of the mechanism behind tryptanthrin‐mediated suppression of skin carcinogenesis in mice

We studied the expression status of cyclin‐D1 and c‐Myc, the target genes of β‐catenin, in the epidermal tissue from respective groups of short‐term experiment by immunohistochemistry. Interestingly, corroborating our earlier results with β‐catenin, both cyclin‐D1 and c‐Myc expression was lower in tryptanthrin‐treated skin (D + P + T) compared with untreated skin (D + P; Figure 5A,B, Figure S5A). Moreover skin tumours from tryptanthrin‐treated animals of the multistage carcinogenesis model showed a down‐regulation of these pro‐tumourigenic proteins (Figure 5C, Figure S5B).

### 3.6 Tryptanthrin exerts potent anti‐cancer activity against epidermoid squamous cell carcinoma cells and suppresses β‐catenin activation

**Figure 6:**
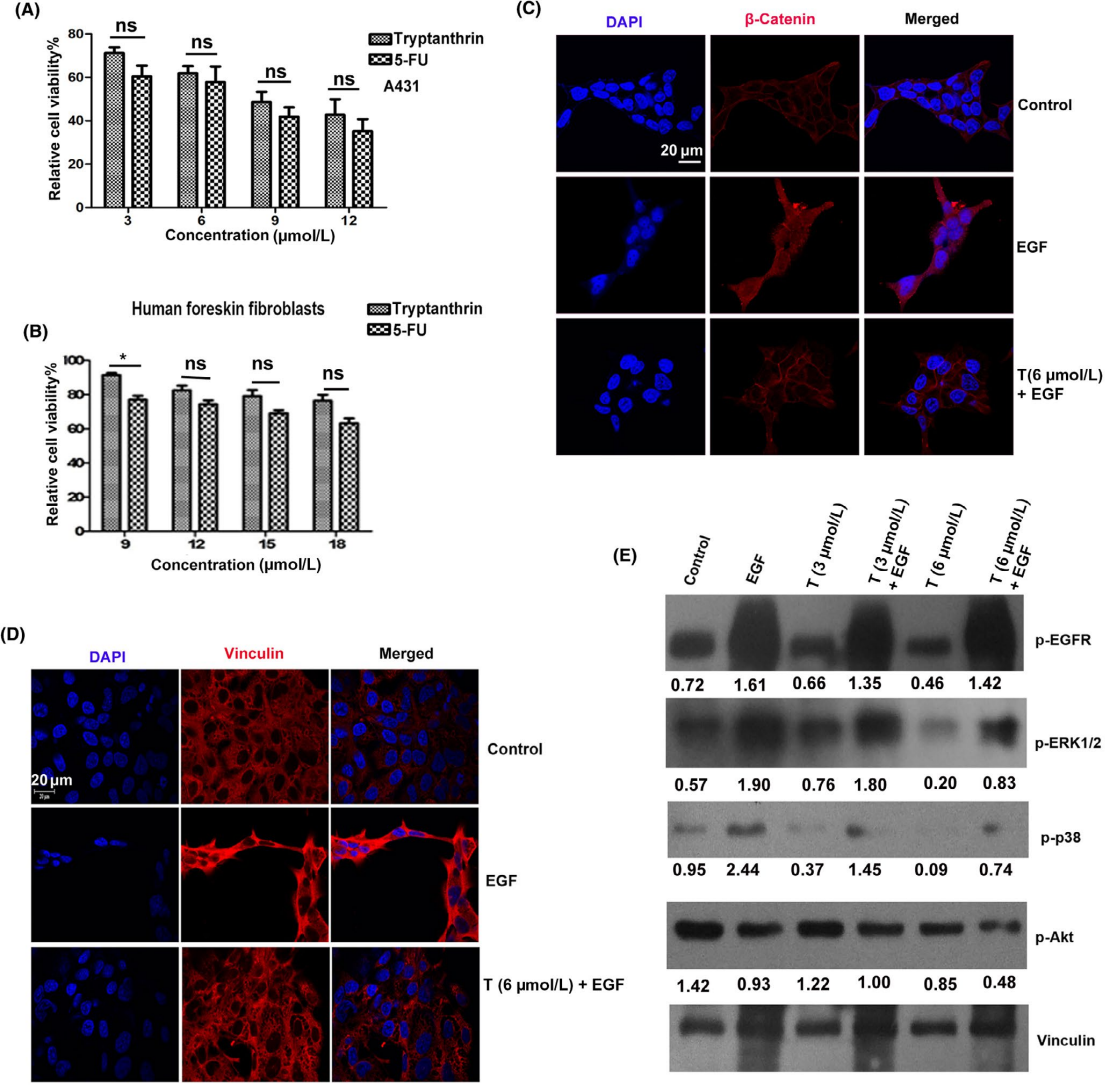
Tryptanthrin exerts potent anti‐cancer activity against epidermoid squamous cell carcinoma cells and suppress β‐catenin activation. A, MTT assay showing extend of cytotoxicity induced by tryptanthrin and 5‐FU in A431 cells. Histograms show average ± SEM. B, MTT assay showing extend of cytotoxicity induced by tryptanthrin and 5‐FU in Human foreskin fibroblasts. C, Confocal image showing the suppression of EGF‐induced β‐catenin activation by tryptanthrin at a concentration of 6 μmol/L in A431 cells. Scale bar, 20 μm. The cells were treated with tryptanthrin 2 h prior to EGF treatment (100 ng/mL), incubated for 24 h for assessing the potency of the compound in inhibiting β‐catenin activation and vinculin rearrangement. Alternatively, cells were treated with tryptanthrin 2 h prior to EGF treatment (100 ng/mL), incubated for 15 min and protein was isolated for assessing the potency of the compound in inhibiting MAPK, Akt activation. D, Confocal Image showing abrogation of EGF‐induced rearrangement of vinculin in A431 cells. Scale bar, 20 μm. E, Immunoblot showing the modulation of EGFR, ERK1/2, P38 and Akt phosphorylation by tryptanthrin in A431 cells

We further assessed whether the anti‐cancer potential of tryptanthrin observed in animal models can be translated to human skin cancer cells. Human epidermoid carcinoma cell line, A431, was treated with tryptanthrin, and the cytotoxicity was compared with that of 5‐FU. Tryptanthrin exhibits IC‐50 at 9 μmol/L, which is comparable to that of 5‐FU (Figure 6A). Interestingly, even at a concentration of 18 μmol/L, tryptanthrin induces only 22% cell death in human foreskin fibroblasts (Figure 6B). Further, tryptanthrin significantly affected the clonogenic potential of A431 and the effect was comparable to that of 5‐FU (Figure S5A). As our previous results indicate a significant role for β‐catenin signalling in regulating the anti‐cancer potential of tryptanthrin, A431 cells pretreated with tryptanthrin were exposed to EGF, a known inducer of β‐catenin signalling,^27^ for 24 hours and subjected to immunofluorescence analysis. Interestingly, tryptanthrin abrogated EGF‐induced activation of β‐catenin even at 6 μmol/L (Figure 6C). EGF‐induced β‐catenin activation leads to cytoskeletal rearrangement characteristic of a mesenchymal phenotype. To assess whether tryptanthrin can abrogate EGF‐induced cytoskeletal rearrangement, A431 cells were treated with EGF for 24 hours with/without tryptanthrin treatment. Tryptanthrin at 6 μmol/L abrogates EGF‐induced cytoskeletal rearrangement as assessed by immunostaining of vinculin (Figure 6D). Furthermore, corroborating the results in mouse skin, tryptanthrin down‐regulates EGF‐induced activation of ERK1/2 and p38 in A431 cells though it failed to inhibit EGF‐induced activation of EGFR (Figure 6E, Figure S7A). We also observed that treatment with EGF and tryptanthrin has suppressed the phosphorylation of Akt, though it did not affect its expression (Figure 6E, Figure S7B). This is consistent with a previous report which describes EGF‐induced attenuation of Akt activation in A431 cells.^28^ To confirm this, we evaluated the phosphorylation status of Akt in response to EGF at different time points, which revealed a consistent decrease in the phosphorylation of Akt (Figure S7B). These results strongly points that tryptanthrin could inhibit β‐catenin signalling by modulating pathways that integrate with β‐catenin signalling. β‐catenin signalling is known to be activated by the canonical Wnt pathway.^29^ Hence, we checked whether tryptanthrin could abrogate Wnt‐induced activation of β‐catenin in A431 cells, by treating the cells with Wnt1a for 24 hours with/without tryptanthrin treatment. However, tryptanthrin did not have much effect in suppressing Wnt‐induced activation of β‐catenin (Figure S8). Considering the extent of suppression of ERK phosphorylation by tryptanthrin, we did molecular docking to check the interaction of tryptanthrin with ERK2. The binding affinity is expressed in terms of Glide gscore. The Glide gscore of tryptanthrin‐ERK2 complex was found to be −6.82 kcal/mol while the selective ERK inhibitor FR180204 scored −9.02 kcal/mol. Thus, the binding affinity of tryptanthrin is inferior to the ERK inhibitor FR180204. Tryptanthrin and FR180204 possess similar binding mode towards the ATP binding pocket of ERK2 (Figure S9, Table.S1).

## 4 DISCUSSION

Over the years, great emphasis has been laid on identifying potent anti‐cancer agents as therapeutics, preventives and sensitizers, from nature. In this study, we report the efficacy of tryptanthrin as a potent anti‐cancer agent against non‐melanoma skin cancer. In mice models, tryptanthrin successfully brings about a drastic reduction in tumour size and multiplicity. The present study shows that the compound impedes skin carcinogenesis by disrupting DMBA/PMA‐induced expansion of hair follicle cells. This is worth noting when we consider previous studies, which report the expansion of mutated cells residing in the hair follicle, as the principal event in DMBA/PMA‐induced skin carcinogenesis.^16^, ^30^ Our result is relevant and raises substantial interest, as several studies have postulated the follicular origin of human basal cell carcinoma (BCC), the most common type of NMSC.^31^, ^32^, ^33^ Moreover, tryptanthrin also suppresses DMBA/PMA‐induced inflammation, a major impetus for carcinogenesis.^34^

Reports on the molecular events operating in human and mouse skin cancer dictate appreciable similarity. Activation of β‐catenin is essential for the expansion of cancer stem cells^35^ and is indispensible for skin carcinogenesis in mice models. Interestingly, dys‐regulation of β‐catenin signalling is seen in human pilomatricoma, BCC and squamous cell carcinoma.^36^ The present study reveals that DMBA/PMA‐induced activation of β‐catenin is effectively restrained by tryptanthrin in the hair follicle cells, thus restricting their expansion and halting subsequent skin tumourigenesis. On the contrary, tryptanthrin did not have any effect on hyperplasia induced by brief application of PMA on normal skin. Application of PMA on normal skin did not show nuclear localization of β‐catenin in the HF or inter‐follicular epidermal (IFE) region, suggesting that β‐catenin signalling is not crucial in induction of hyperplasia. This is in agreement with the previous report that hyperproliferation of IFE cells does not depend on activation of β‐catenin.^19^

The critical role of proliferative and survival pathways such as MAPKs, Akt and STAT3 in DMBA/PMA‐induced skin tumours^22^, ^37^, ^38^ and human cutaneous SCC and BCC is well established.^39^, ^40^, ^41^ Activation of β‐catenin is positively correlated to a spike in MAPK and Akt signalling.^23^, ^42^, ^43^, ^44^, ^45^ ERK1/2‐mediated transactivation of β‐catenin in human cutaneous SCC is well established.^43^ Similarly, activation of ERK1/2 and p38 has been found to be upstream to β‐catenin signalling and is reported to be momentous for tumourigenesis in mouse models.^22^, ^23^ In the present work, dissection of the signalling events discovered that tryptanthrin could mitigate DMBA/PMA‐induced activation of proliferative signals in mouse skin with ERK1/2 activation showing maximum attenuation. Phosphorylation of c‐Jun, a component of AP‐1 transcription complex downstream to ERK1/2,^46^ was significantly less in the nuclear extracts isolated from the tissues of tryptanthrin‐treated animals. Interestingly, DMBA/PMA treatment induced phosphorylation of ERK and Akt, mainly in hair follicle cells. This is in concordance to the previous findings that demonstrate preferential activation of MAPKs and Akt in cancer stem like cells.^47^, ^48^ Notably, application of tryptanthrin abrogated DMBA/PMA‐induced phosphorylation of these proteins in the hair follicle cells. This is in agreement to our observation, which demonstrates a significant reduction in nuclear translocation of β‐catenin in the HF cells of tryptanthrin‐treated animals. However, we observed only a moderate increase in phosphorylated ERK in hyperplastic skin, compared with the greater extent of ERK phosphorylation in DMBA/PMA‐treated skin. It has been reported that hERK1 knockout mice exhibits a partial reduction in hyperproliferation of epidermis in response to PMA application.^49^ Though tryptanthrin could suppress PMA‐induced phosphorylation of ERK1/2, it did not suppress epidermal hyperplasia suggesting that a transient and incomplete inactivation of ERK1/2 and Akt is insufficient in attenuating PMA‐induced epidermal hyperplasia. It must be noted that application of PMA alone could not induce activation of β‐catenin in epidermis and hair follicles, suggesting that activation of ERK1/2 and Akt could be determinants of β‐catenin activation in skin. Moreover, *CYCLIN‐D1* and *C‐MYC*,^50^ the transcriptional targets of β‐catenin, which are widely implicated in carcinogenesis,^51^, ^52^ showed low expression in skin tumours of tryptanthrin‐treated mice. Thus, we conclude that tryptanthrin suppresses skin carcinogenesis by suppressing inflammation and hindering β‐catenin and the associated signalling pathways in the hair follicle cells, thus impeding their expansion and consequent tumour development (Figure 5D).

The study illustrates that tryptanthrin induces significant toxicity against human epidermoid carcinoma cell line, A431 while being non‐toxic to normal skin cells. Treatment with EGF caused the translocation of β‐catenin from the membrane to the cytoplasm and to some extent, to the nucleus. Accumulation of β‐catenin in the cytoplasm leads to its nuclear translocation.^53^ Corroborating our results obtained from the animal model, tryptanthrin suppresses the activation of β‐catenin and associated proliferative signals in these cells too.

Thus the study demonstrates with mechanism based evidence that tryptanthrin is a potent molecule against NMSC and recommends its candidature in clinical trials.

## CONFLICT OF INTEREST

The authors report no potential conflicts of interest.

## AUTHOR CONTRIBUTIONS

MSG, SVB and RJA designed the experiments. MSG, VVA, ANA and APR carried out the experiments. MSG, RJA, SVB, SS, APR and SC analysed the data. MSG, RJA, SVB, SC, ANA and VVA prepared the manuscript. RJA conceived the study. All authors read and approved the final manuscript.

## Data Availability

The data that support the findings of this study are available from the corresponding author upon reasonable request.
