# Supplementary material for: Pre‐clinical evidences for the efficacy of tryptanthrin as a potent suppressor of skin cancer
Source: Cell Prolif. 2019 Oct 30;53(1):e12710. doi: 10.1111/cpr.12710 (PMC6985671; doi:10.1111/cpr.12710)
Supplement: Supplementary file 1 [file CPR-53-e12710-s001.docx]

| 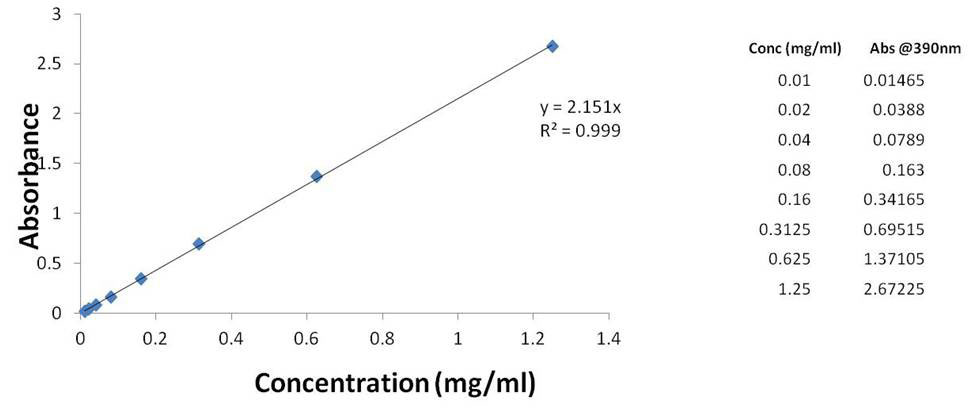  **Figure S1 Standard graph showing the absorbance of different concentrations of tryptanthrin.**  Encapsulation efficiency of tryptanthrin was calculated using the formula (Final drug concentration/Initial drug concentration)×100**.**  **2. A**  **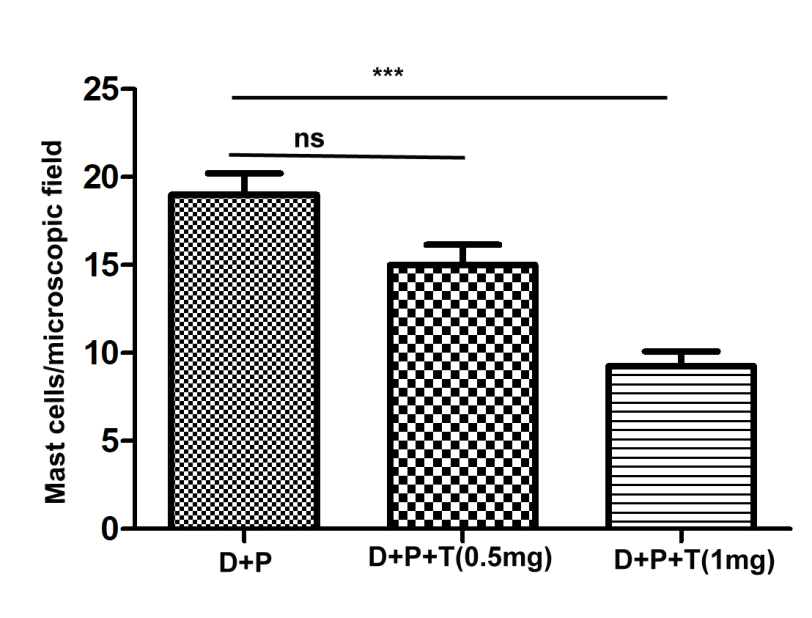**  **B**  **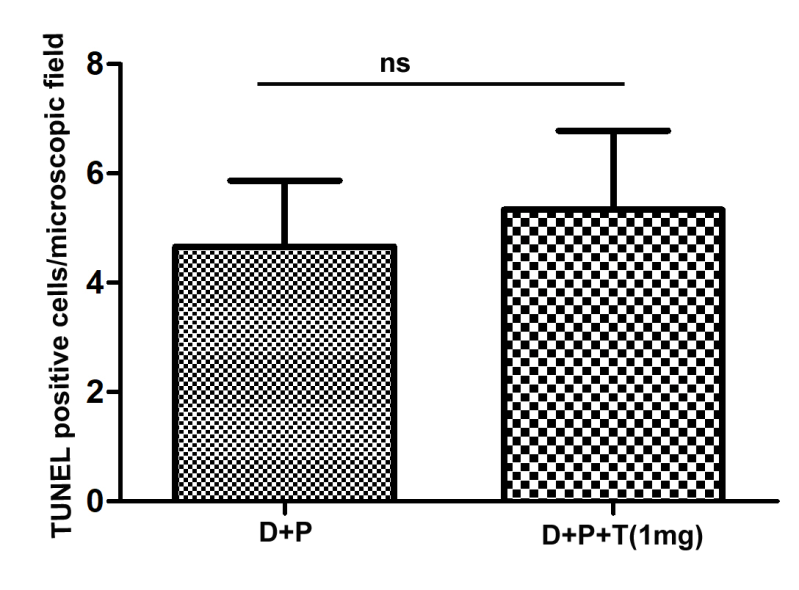**  **C**  **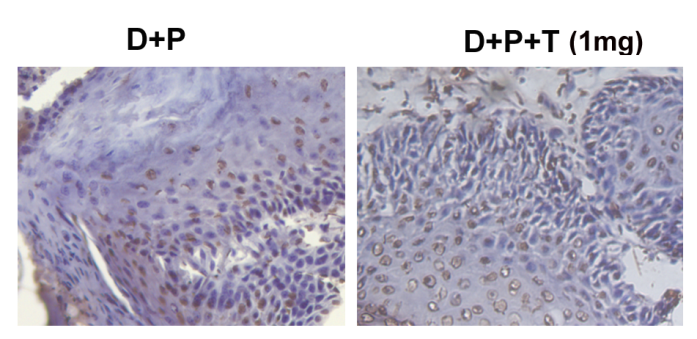 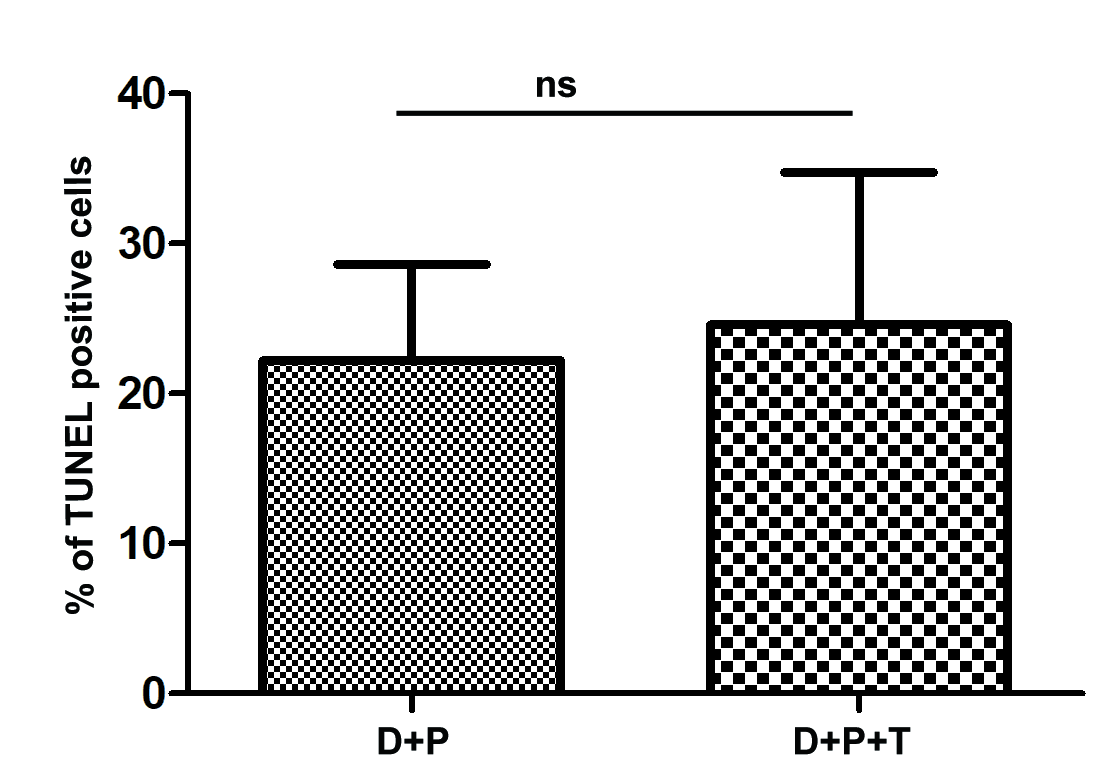**  **Figure S2**  **2. Tryptanthrin significantly suppress DMBA/PMA induced inflammation while it doesn’t induce apoptosis . A.** Graph indicating toluidine positive cells/microscopic field **B.** Graph indicating TUNEL positive cells/microscopic field in the respective groups. **C.** TUNEL positive cells in tumors from untreated and treated groups (n=3). Graph indicating TUNEL positive cells/microscopic field of tumors isolated from tryptanthrin treated and untreated groups (n=3).    **3**  **A** 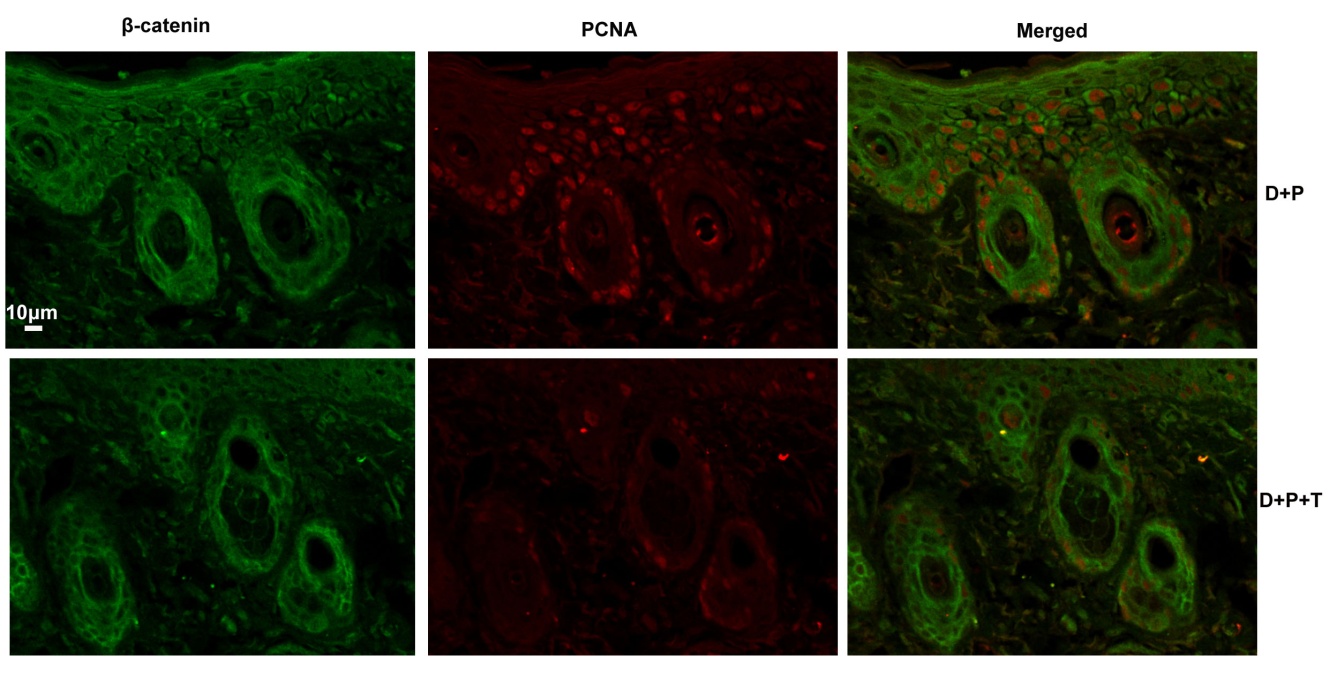  **B**  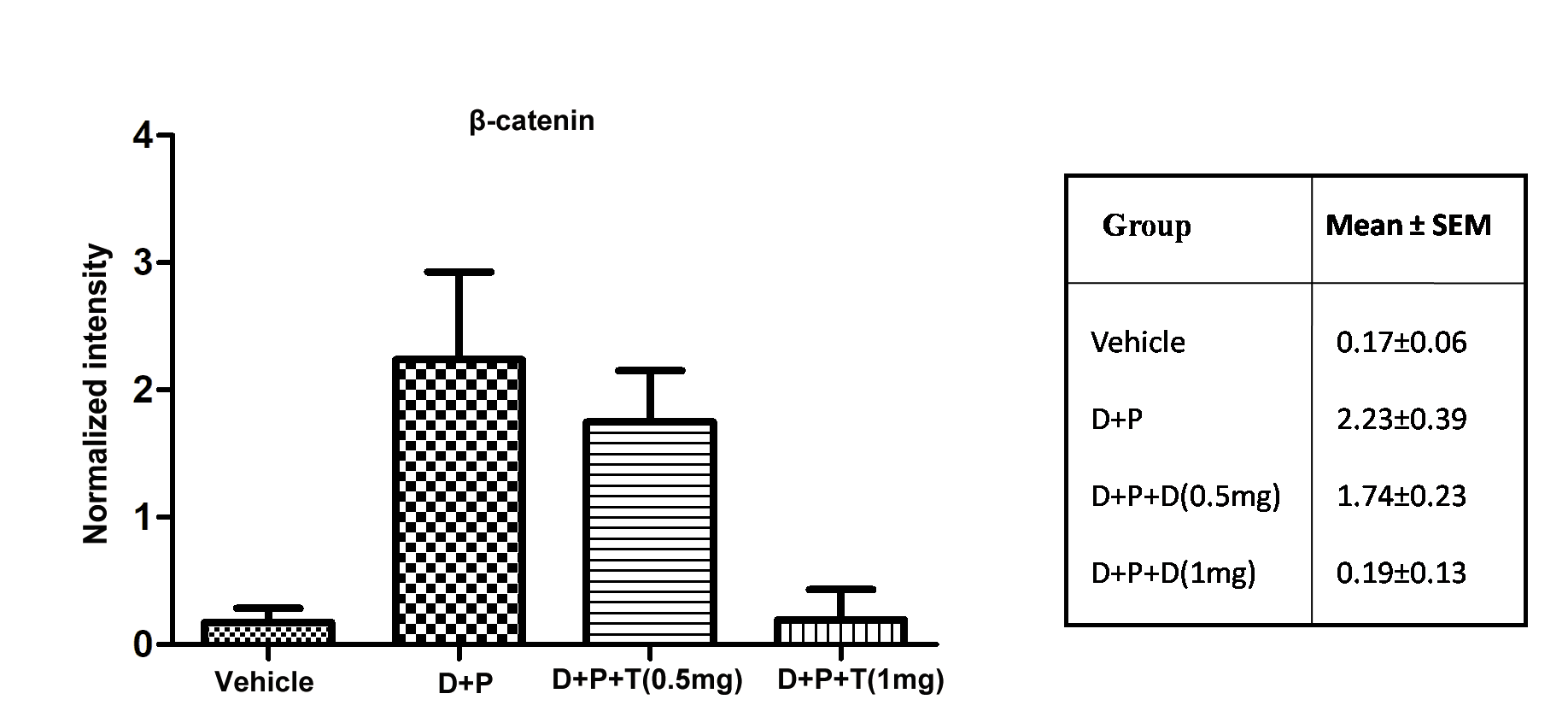  **C**  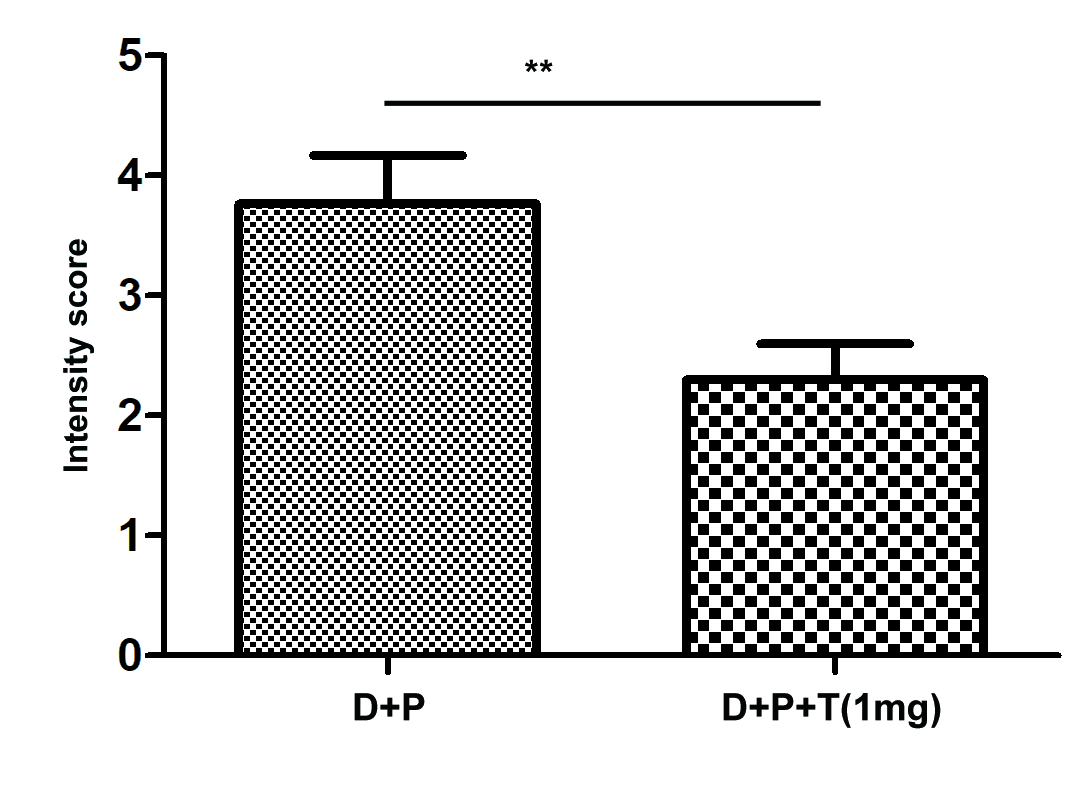  **β-catenin**  **Figure S3**  **Tryptanthrin suppresses DMBA/PMA induced β-catenin activation and hair-follicle cell proliferation.** **A.** Double immunoflourescent staining of β-catenin and PCNA in skin from tryptanthrin treated and untreated groups. **B.** Normalized intensity of β-catenin in the respective groups as assessed by immunoblot analysis (n=3). **C.** Graph showing intensity of nuclear β-catenin in tumors from untreated and treated groups.  **4. A**  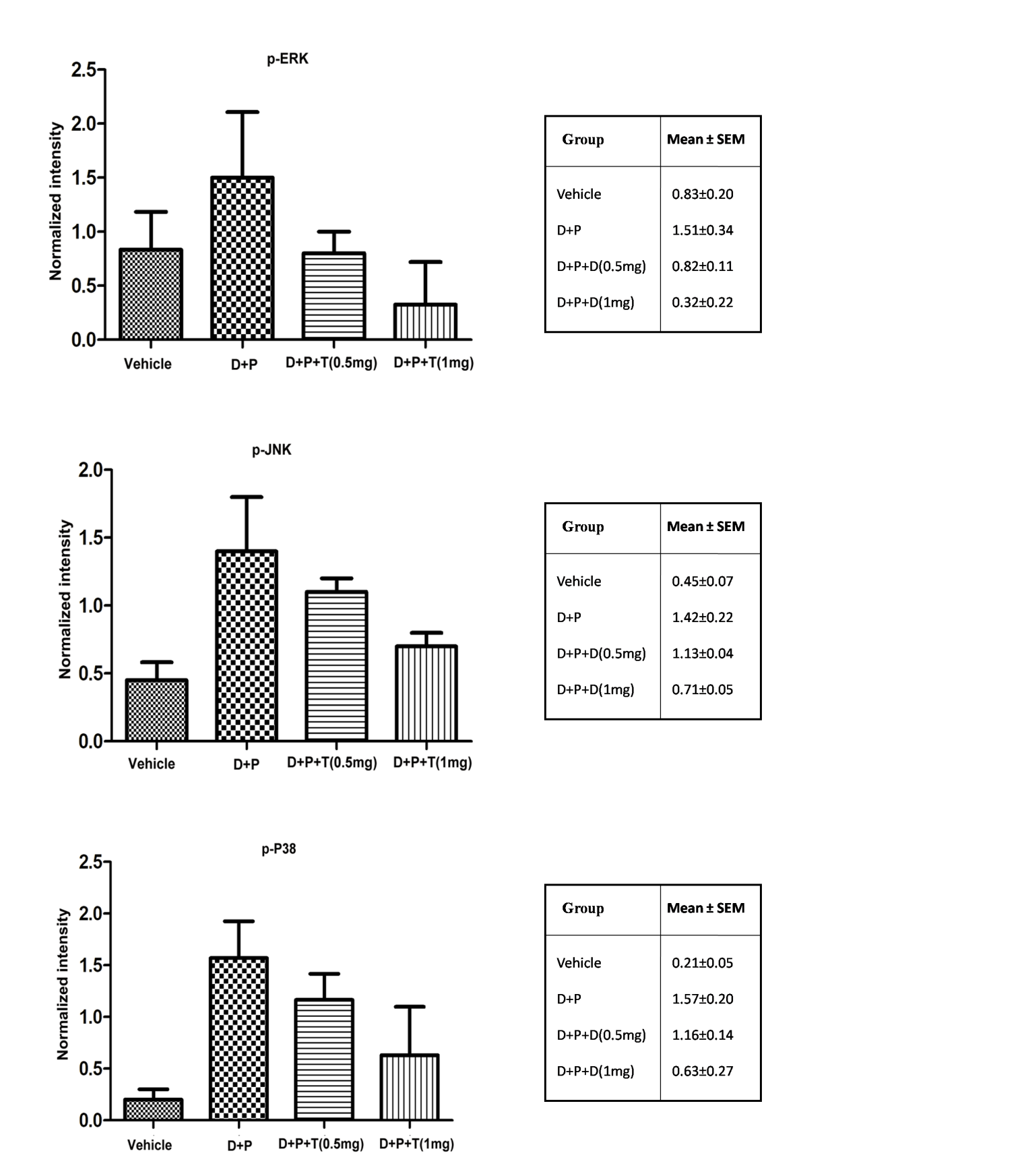  **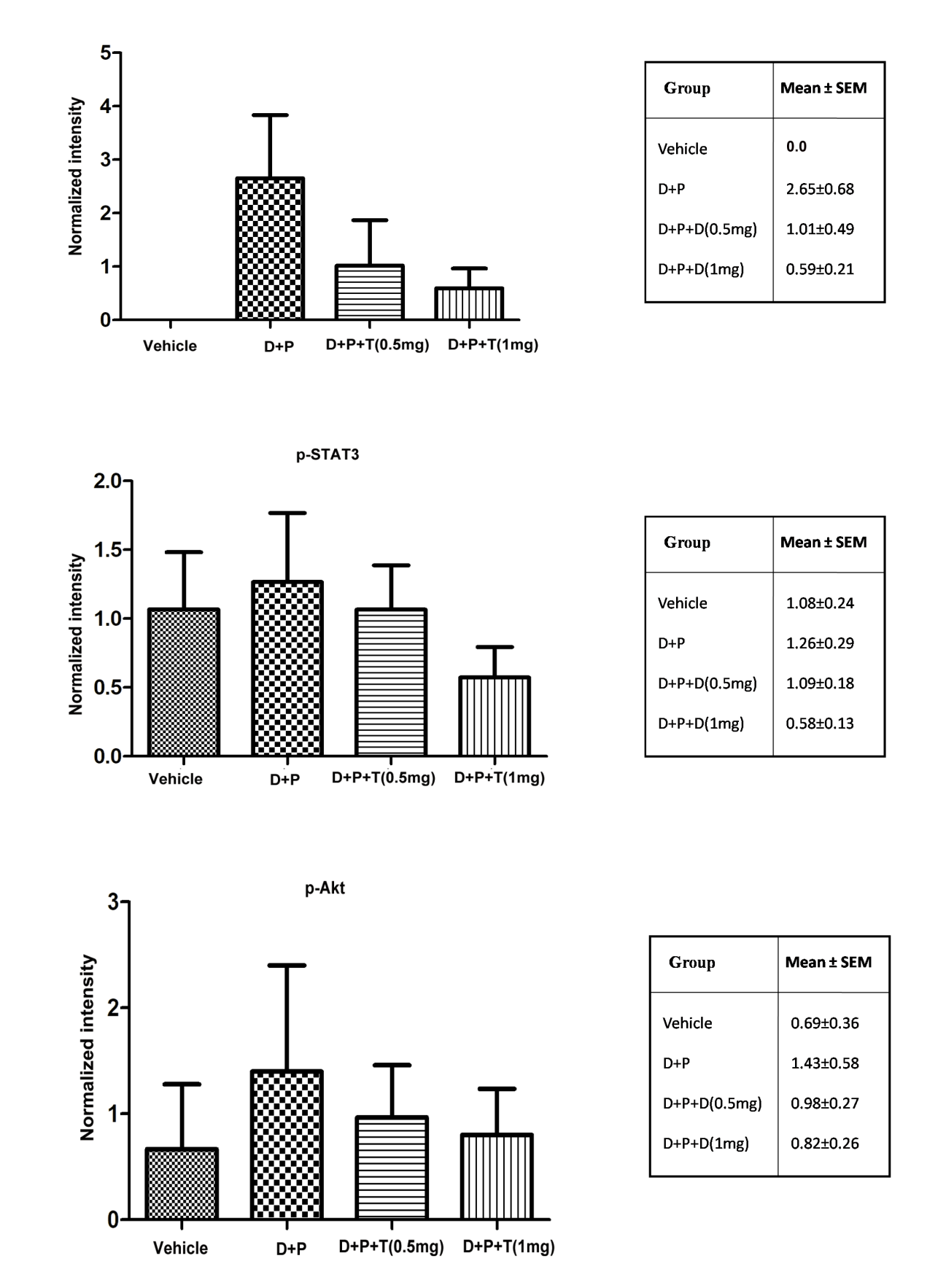**  **p-c-JUN**  **B.**  **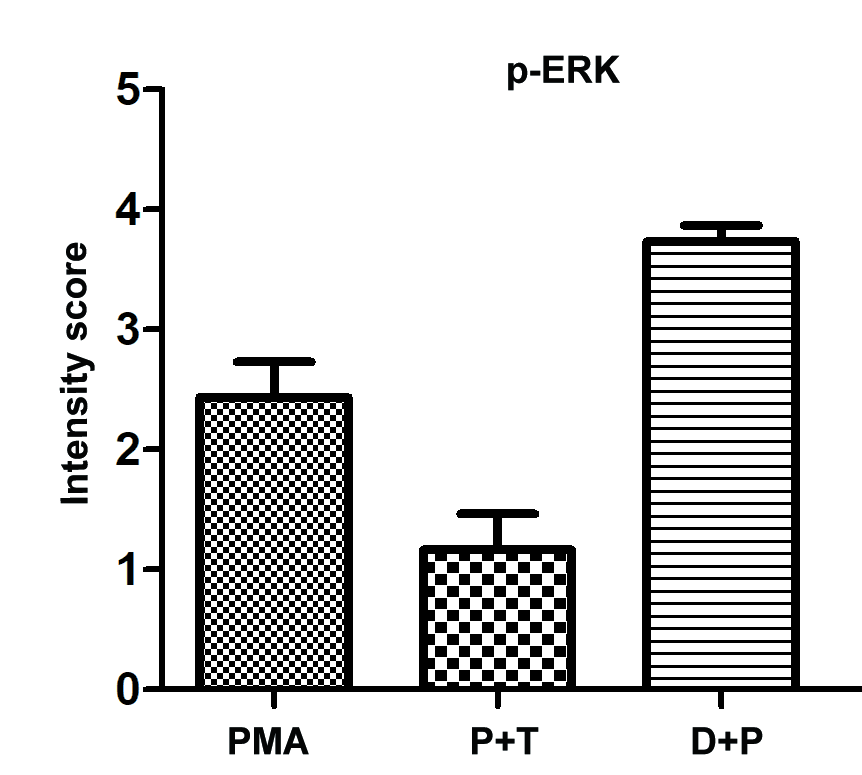 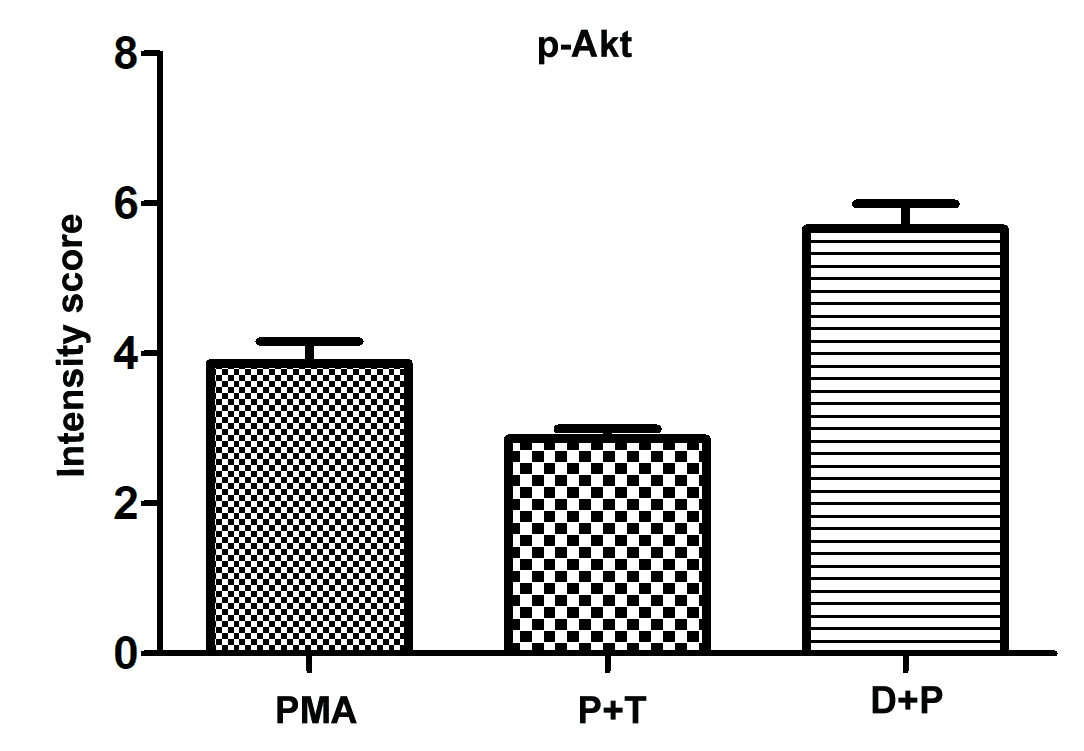**  **Figure S4**  **Tryptanthrin suppresses DMBA/PMA induced phosphorylation of proliferative and survival signals**. **A.** Graph showing the relative phosphorylation levels of proteins in the respective groups as assessed by immunoblot analysis. Intensity of phosphoproteins was normalized to the intensity of loading controls. **B.** Graph showing the intensity score of p-ERK1/2 and p-Akt in the respective groups as assessed by immunohistochemical analysis.  **5**  **A.**  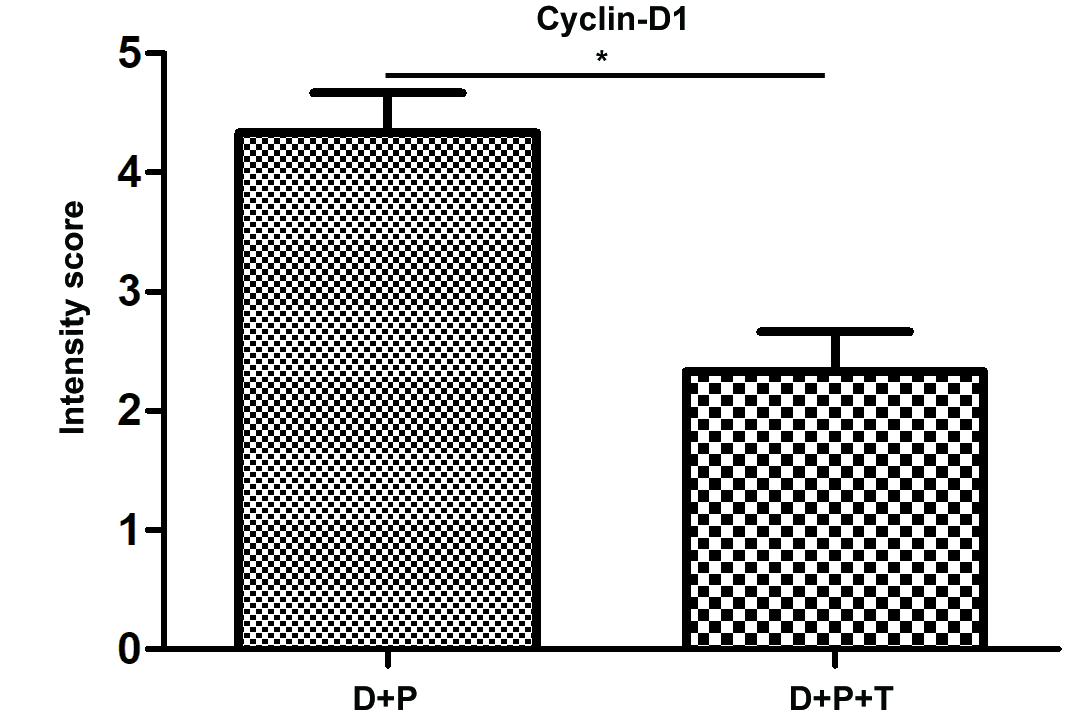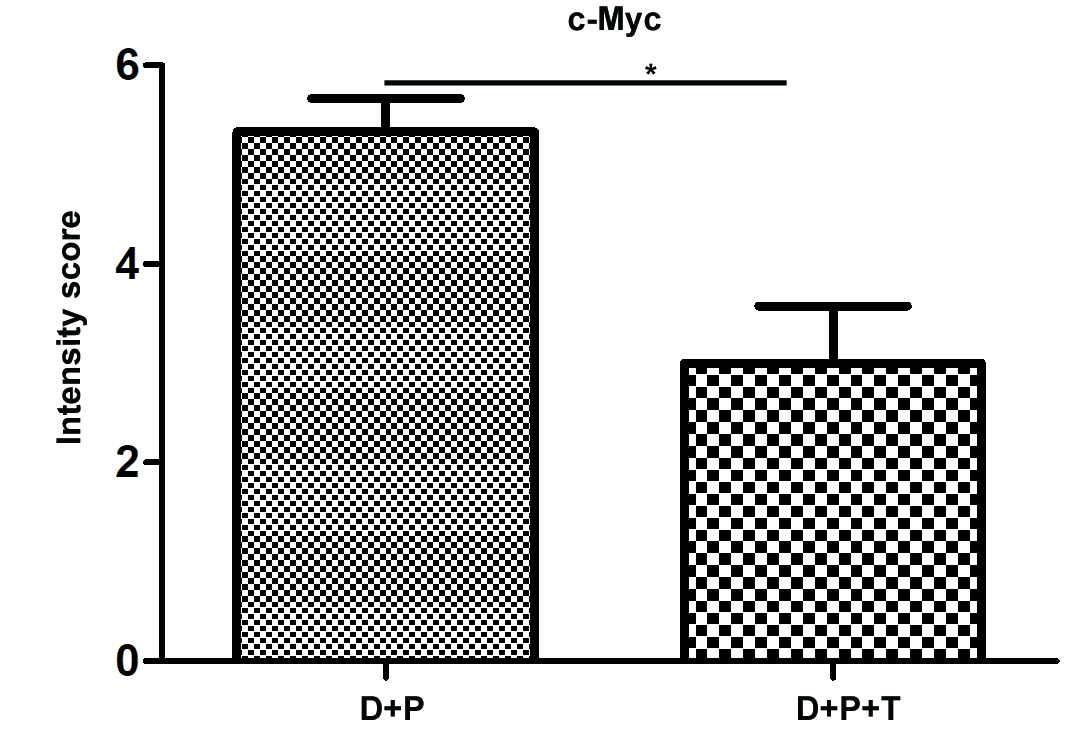  **B.**  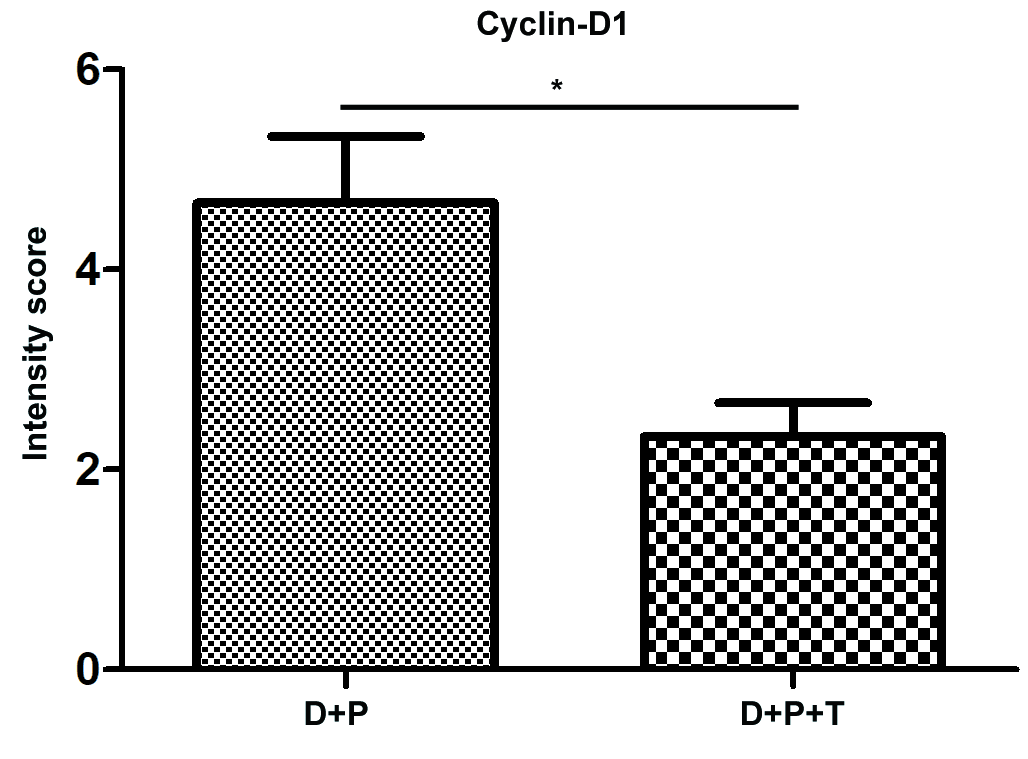 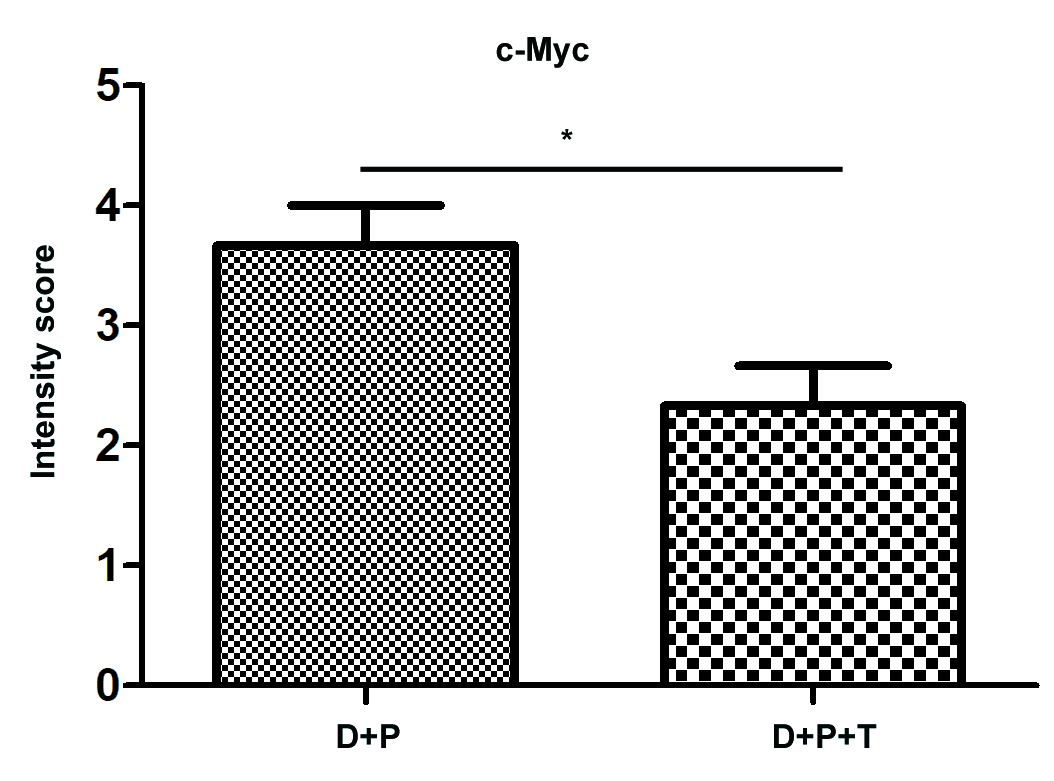  **Figure S5**  **Tryptanthrin suppresses the expression of cyclin-d1 and c-myc**. **A.** Graph showing the intensity score of cyclin-D1 and c-myc in skin from the respective groups as assessed by immunohistochemical analysis (n=3). **B.** Graph showing the intensity score of cyclin-D1 and c-myc in tumour from the respective groups as assessed by immunohistochemical analysis (n=3).  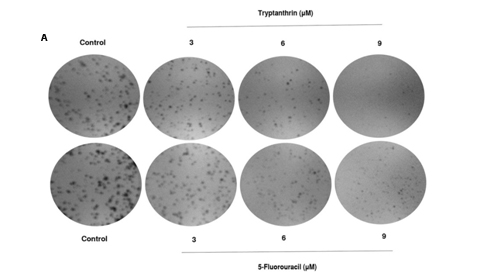  **Figure S6**  **Tryptanthrin suppresses the clonogenic potential of A431 cells**. Clonogenic assay showing the potential of tryptanthrin and 5-FU in inhibiting colony formation of A431 cells.  **A.**  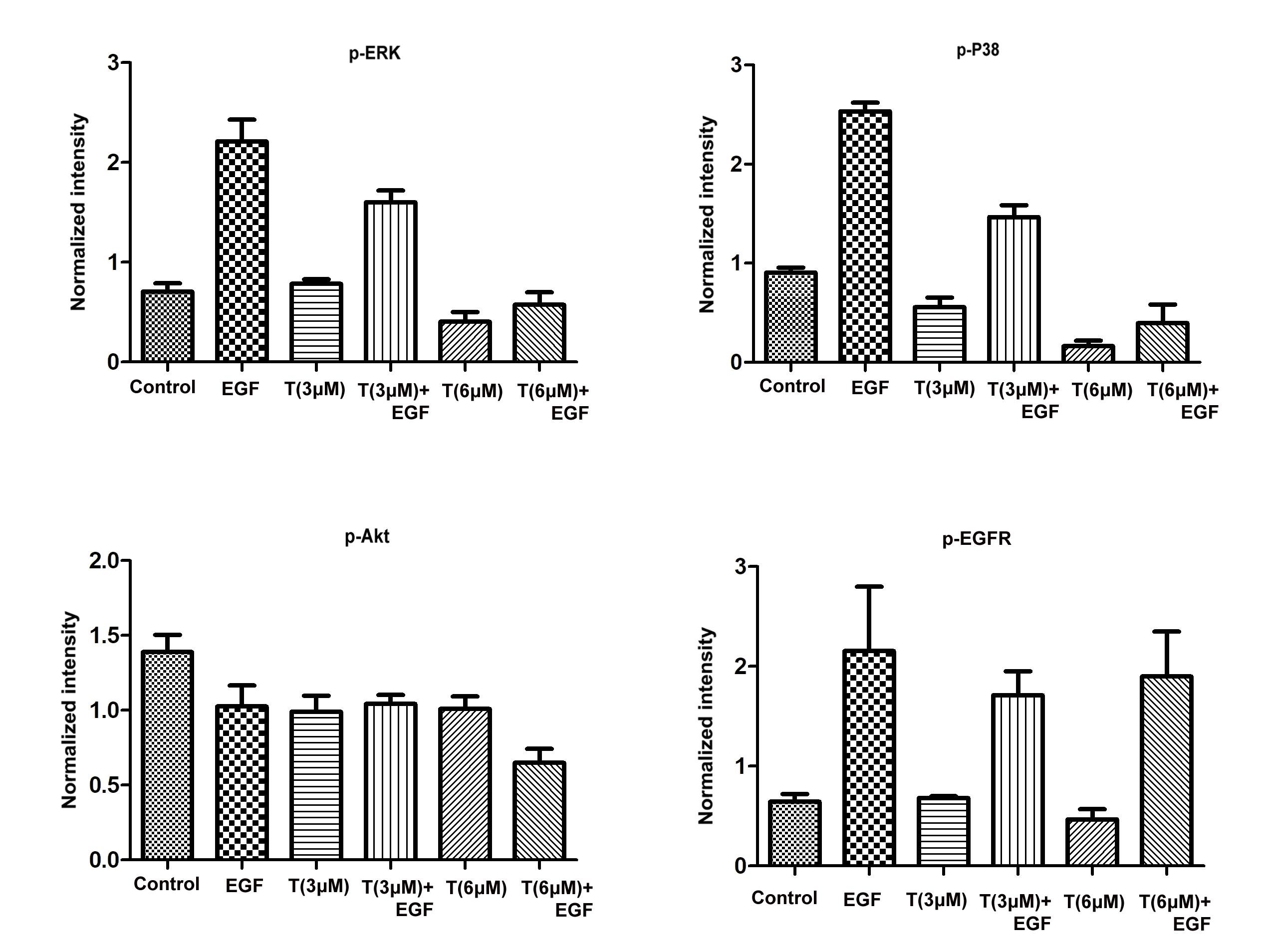  **B.**  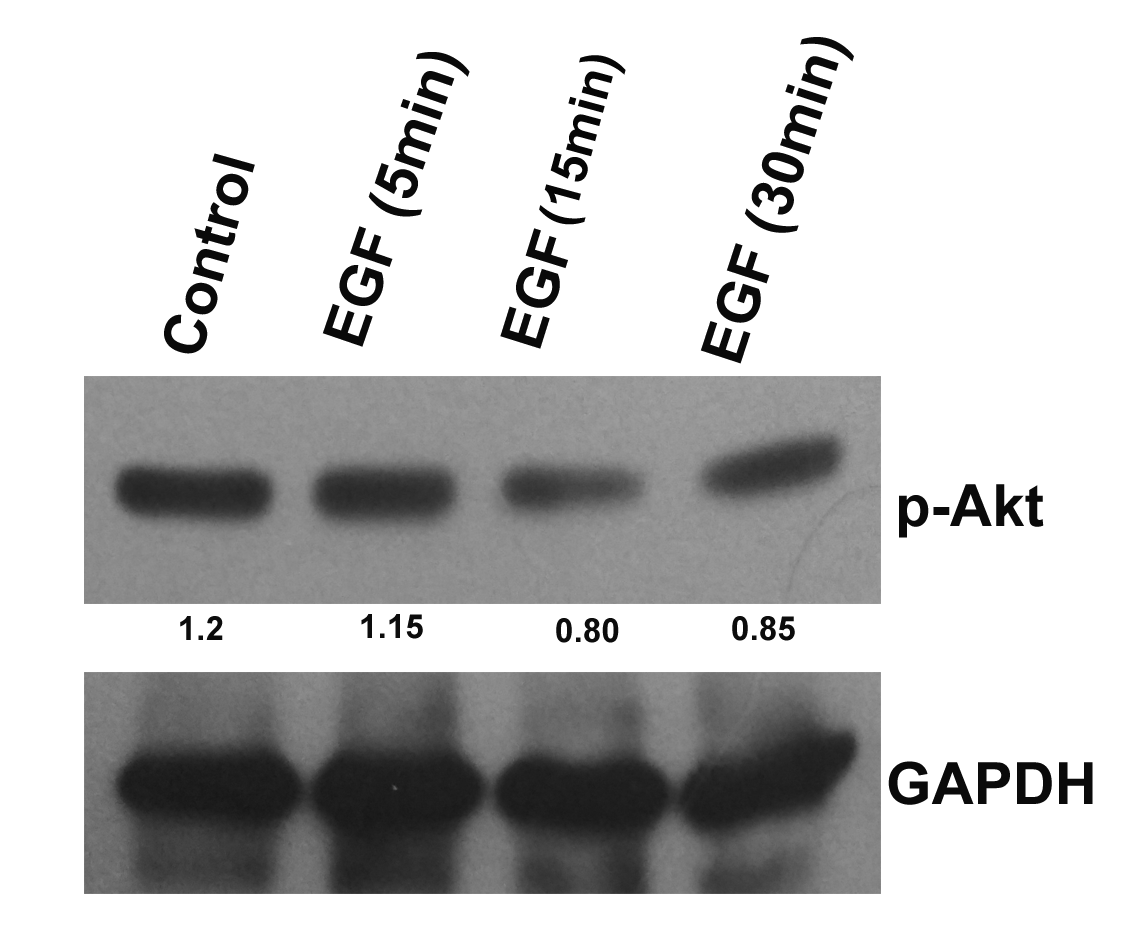 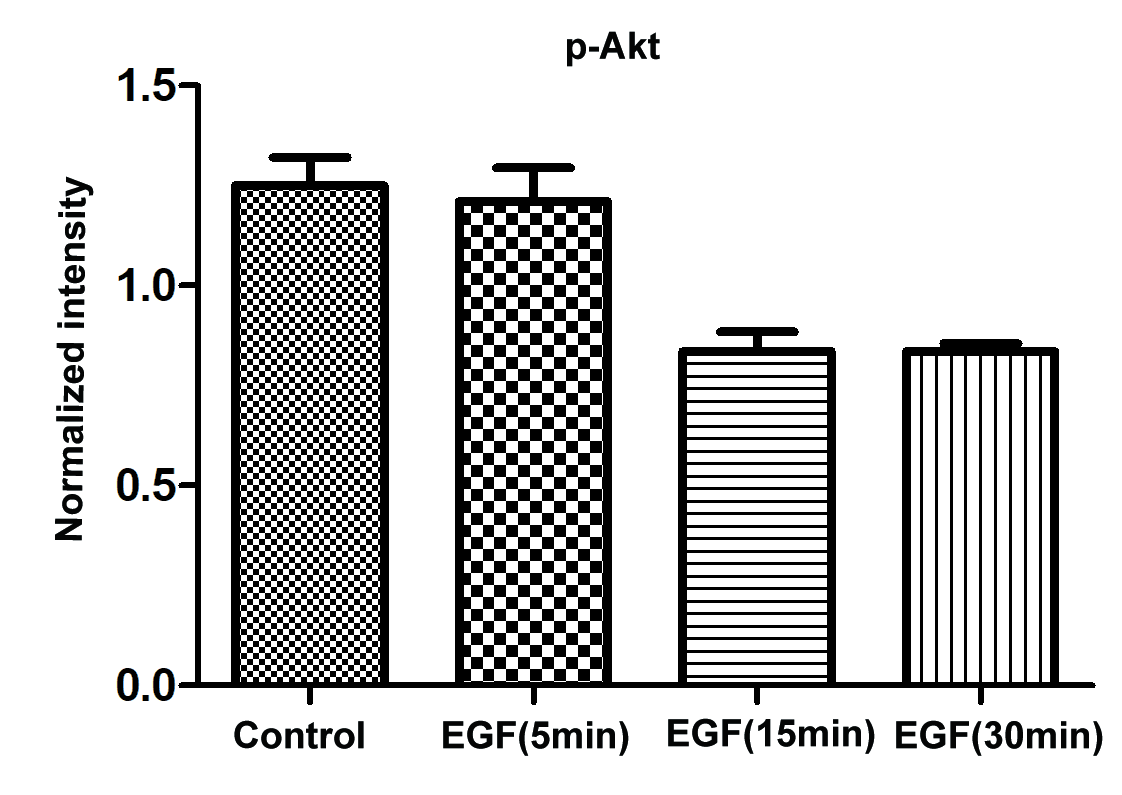b.  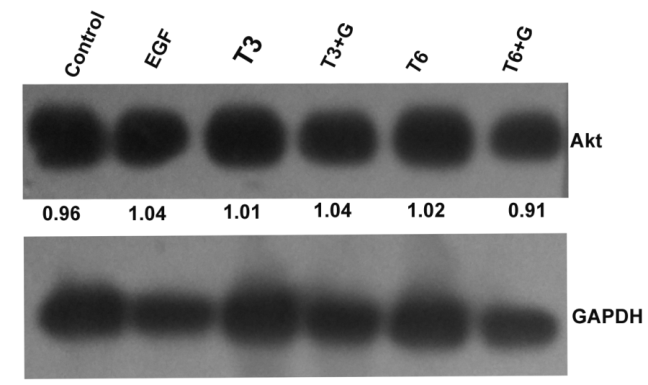  **Figure S7**  **Tryptanthrin suppresses EGF induced activation of proliferative signals.** **A.** Graph showing the tryptanthrin mediated modulation of EGF induced phosphorylation of proliferative signals (n=3). Normalized intensity plotted as Mean±SEM. **B**. Immunoblot analysis of EGF induced modulation of Akt phosphorylation at different time points. Graph shows the normalized intensity from two independent experiments. **C**. Immunoblot analysis of total Akt in cells treated with tryptanthrin and EGF. Intensity values were normalized to loading control.  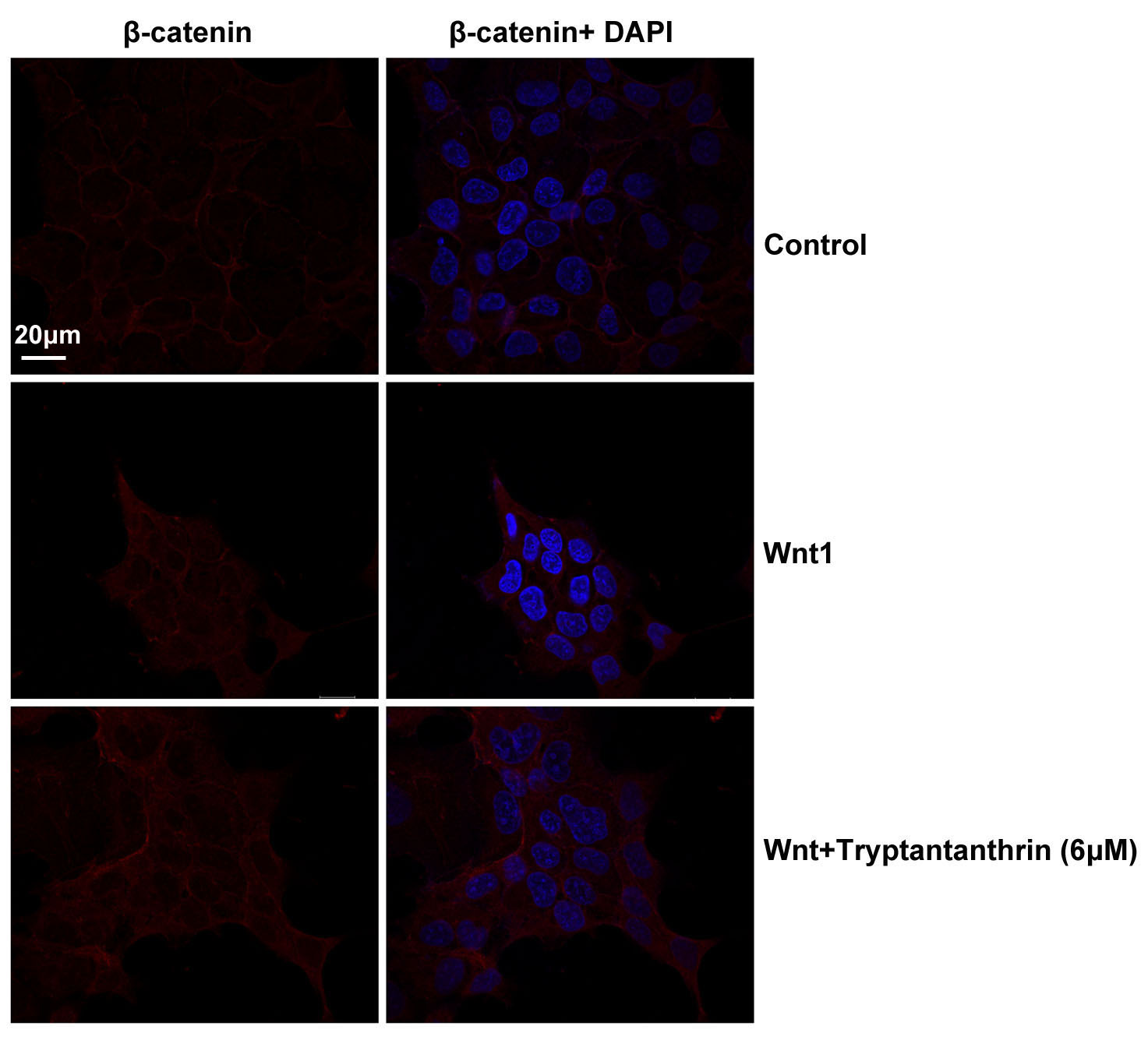  **Figure S8**  **Tryptanthrin does not suppress Wnt1a induced activation of β-catenin in A431 cells**. Tryptanthrin was treated 2h prior to treatment with 200ng/ml of Wnt1a and the cells were incubated for 24h after which it was processed for immunoflourescence analysis and the subcellular localization of β-catenin was assessed by confocal microscopy. Scale bar,20μm  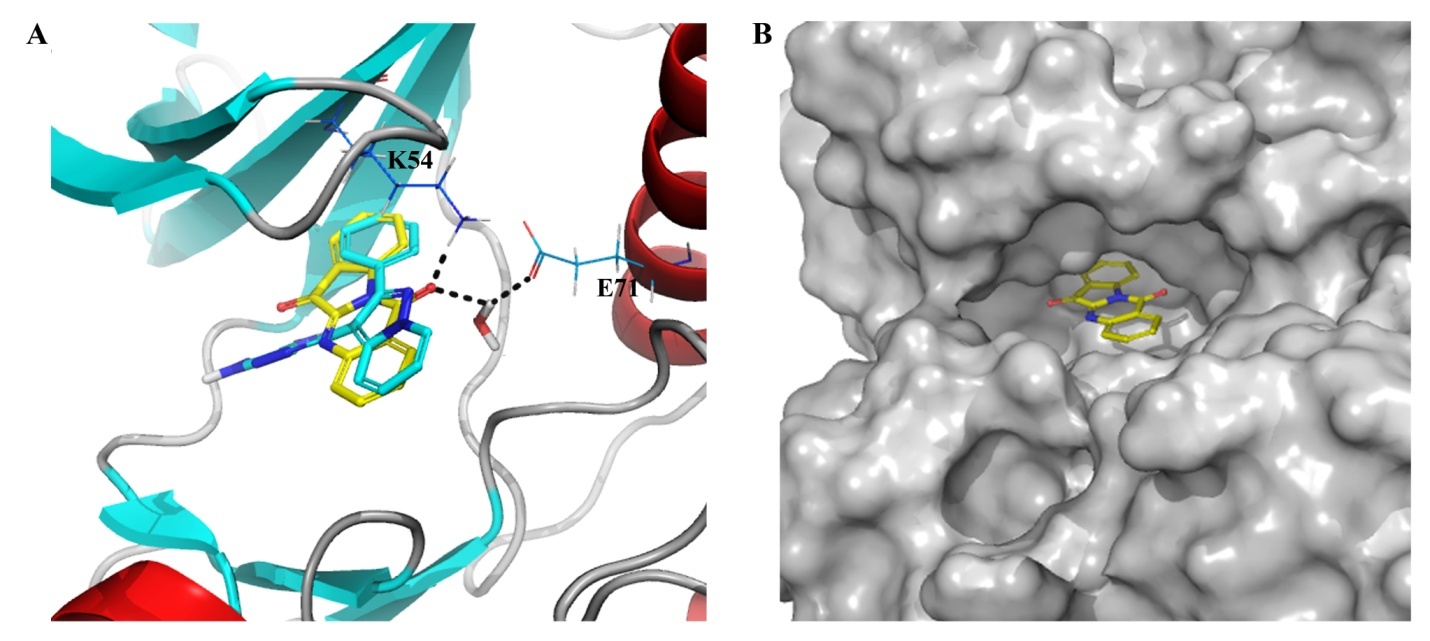  **Supplementary Figure S. 9.** Comparative binding orientation of tryptanthrin (yellow stick) with the selective ERK inhibitor FR180204 (cyan stick) in the ATP binding pocket of ERK2. Tryptanthrin binds the ATP binding pocket of ERK2 by forming H-bond interaction with K54 and a water mediated H-bond with E71. **B.** Surface representation of the ATP binding pocket of ERK2 with the docked tryptanthrin (yellow stick).  **Supplementary Table 2.** Molecular docking scores and binding free energies of compounds   \| **Compound** \| **Glide gscore (kcal/mol)** \| **Binding energy (kcal/mol)** \| \| --- \| --- \| --- \| \| Tryptanthrin \| -6.82 \| -32.29 \| \| FR180204 \| -9.02 \| -44.50 \| |
| --- | --- | --- | --- | --- | --- | --- | --- | --- | --- |
|  |
